# Group decision-making with Fermatean fuzzy soft expert knowledge

**DOI:** 10.1007/s10462-021-10119-8

**Published:** 2022-01-09

**Authors:** Muhammad Akram, Ghous Ali, José Carlos R. Alcantud, Aneesa Riaz

**Affiliations:** 1 Department of Mathematics, University of the Punjab, New Campus, Lahore, Pakistan; 2 Department of Mathematics, Division of Science and Technology, University of Education, Lahore, Pakistan; 3 BORDA Research Unit and IME, University of Salamanca, 37007 Salamanca, Spain

**Keywords:** Fermatean fuzzy soft expert set, Algorithm, Solar panel system, MCGDM

## Abstract

With the rapid growth of population, the global impact of solar technology is increasing by the day due to its advantages over other power production technologies. Demand for solar panel systems is soaring, thus provoking the arrival of many new manufacturers. Sale dealers or suppliers face an uncertain problem to choose the most adequate technological solution. To effectively address such kind of issues, in this paper we propose the Fermatean fuzzy soft expert set model by combining Fermatean fuzzy sets and soft expert sets. We describe this hybrid model with numerical examples. From a theoretical standpoint, we demonstrate some essential properties and define operations for this setting. They comprise the definitions of complement, union and intersection, the OR operation and the AND operation. Concerning practice in this new environment, we provide an algorithm for multi-criteria group decision making whose productiveness and authenticity is dutifully tested. We explore a practical application of this approach (that is, the selection of a suitable brand of solar panel system). Lastly, we give a comparison of our model with certain related mathematical tools, including fuzzy and intuitionistic fuzzy soft expert set models.

## Introduction

Till date, action-oriented research has produced many competing models for the representation of imprecisely defined situations, which mean uncertain situations such as those associated with a shortage of compact and accurate knowledge. These models include probability theory, fuzzy sets (Zadeh 1965), rough sets (Pawlak 1982) or soft sets (Molodtsov 1999). Here we focus on the track of thought initiated by fuzzy set theory which has proven its influence in many areas (Alcantud and Calle 2017; Alcantud et al. 2018; Kahraman and Kaya 2010). A fuzzy set only embodies a membership part, thus it was soon realized that this could make the applied studies overlook important facts regarding the non-membership scores of the alternatives under consideration. Bearing in mind this setback, Atanassov (1986) proposed the intuitionistic fuzzy model. It is a fairly natural generalization of fuzzy set (FS) theory: in an intuitionistic fuzzy set (IFS), all the objects of the universe are characterized by both their membership and non-membership degrees, and their sum is always bounded by 1. However if the experts submit estimates whose sum is greater than one in at least one case, then decision-making based on IFSs will no longer be of use. Therefore to remove this deficiency, a yet more general concept was developed by Yager (2013), namely, the Pythagorean fuzzy set (PFS). The PFS model has soon gained much attention from many experts and researchers. For instance, Yager and Abbasov (2013) discussed about connections between the Pythagorean membership grades (PMGs) and complex numbers. Concerning applications, Peng et al. (2020) designed a Pythagorean fuzzy multi-criteria decision method for fifth generation (5G) wireless industry evaluation. Peng and Selvachandran (2019) is a good survey of the state of the art of the research about PFSs. But also from a theoretical standpoint, this model raised a debate as to how far one can go to represent data within a similar framework. In this direction, Senapati and Yager (2020) put forward the notion of Fermatean fuzzy sets (FFSs) as a further extension of the IFSs and PFSs. In a FFS, the cubic sum of membership and non-membership values of an object are bounded by 1. Numerous investigations have originated with FFSs. For instance, Liu et al. (2019a) developed the notion of Fermatean fuzzy linguistic term set. Senapati and Yager (2019) discussed some new operations over Fermatean fuzzy numbers. Decision-making analysis based on Fermatean fuzzy Yager aggregation operators (with an application in coronavirus disease 2019 (COVID-19) testing facilities) was studied by Garg et al. (2020). Yang et al. (2020) discussed the differential calculus of Fermatean fuzzy functions. Shahzadi and Akram (2021) developed a novel decision-making concept to select an antivirus mask under Fermatean fuzzy soft information. Additionally, Akram et al. (2020) proposed a novel decision-making framework for the selection of an effective sanitizer to reduce COVID-19 under a Fermatean fuzzy environment. Liu et al. (2019b) proposed the concept of distance measure for Fermatean fuzzy linguistic term sets based on linguistic scale function. This is illustrated by their use in the development of TODIM (TOmada de Decisão Interativa e Multicritério/Interactive Multicriteria Decision-Making) and TOPSIS (Technique for Order of Preference by Similarity to Ideal Solution) methods. Other more general models like spherical or *T*-spherical fuzzy sets extend the scope of applicability of the TOPSIS method even further (Munir et al. 2021).

Another source of inspiration for our approach is soft set theory (Molodtsov 1999), that originally addresses situations without a numerical component. Objects are fully portrayed by a compound of attributes and each of them gives an approximate partial description. With this feature, it is drastically different from fuzzy (or intuitionistic, Pythagorean, Fermatean, ...) specifications. After the production of soft sets, many researchers and experts were actively engaged in developing the abilities of this theory. For example, Ali et al. (2009) soon presented additional properties of soft sets. In addition, Maji et al. (2002) were the first who discussed decision-making algorithm based on soft sets. Then other authors proved its significance in different areas, (v., Feng et al. 2010; Feng and Li 2013; Santos-Buitrago et al. 2019). Although soft and fuzzy theories are different, a hybrid tool can be generated by their combination as shown by Maji et al. (2001) who proposed the applicable concept of fuzzy soft set. In fact hybridization techniques are commonly used to acquire increasingly general knowledge and solve new real-world situations (Alcantud et al. 2017; Garg and Arora 2021; Xiao et al. 2009; Ali and Ansari 2021; Zhu et al. 2012; Zhu and Xu 2014). In relation with the subject of our study, Salsabeela and John (2021) presented a TOPSIS method for the hybrid model called Fermatean fuzzy soft set.

A different line of improvement came from Alkhazaleh and Salleh (2011) who introduced the concept of soft expert sets (SESs). They have an edge on the previous models, namely, their inbuilt ability to incorporate multi-expert opinions. These authors studied a decision-making application of SESs. Afterwards, Alkhazaleh and Salleh (2014) developed a hybrid structure called fuzzy SESs. With the production of SESs, several researchers have presented many new hybrid models (Adam and Hassan 2016; Al-Qudah and Hassan 2017; Qayyum et al. 2016; Mishra et al. 2021; Keshavarz-Ghorabaee et al. 2020; Aydemir and Gunduz 2020). For a quick overview of the merits and disadvantages of major existing decision-making methods that have an important role in this study, the readers are referred to Table 1.

Motivated by these facts, in this article we develop a novel hybrid structure, namely, the Fermatean fuzzy SES model that combines FFSs and SESs. We describe it with the help of numerical examples. Furthermore, we demonstrate some of its essential properties and we set forth set-theoretical operations comprising complement, union and intersection, plus the OR and AND logical operations. With this background we explore a practical application of the suggested approach in MCGDM (that is, the selection of the brand of a solar panel system for purchase). An algorithm stated in the context of the proposed model guarantees its proficiency and authenticity. In this paper, we also provide a comparison of our model with related mathematical tools.

**Table 1:** Summary of existing models and decision-making approaches

Researcher(s)	Mathematical Model	Advantages	Limitations	MCGDM
Atanassov (1986)	Intuitionistic fuzzy sets or IFSs	It deals with belongingness and non-belongingness functions	It cannot deal with belongingness and non-belongingness degrees whose sum is above 1	No
Yager (2013)	Pythagorean fuzzy sets or PFSs	It increases the space of belongingness and non-belongingness evaluations	It cannot deal with belongingness and non-belongingness degrees whose sum of squares is above 1	No
Senapati and Yager (2020)	Fermatean fuzzy sets or FFSs	It provides more flexibility to the belongingness and non-belongingness evaluations as compared to IFSs and PFSs	It cannot deal with belongingness and non-belongingness degrees whose sum of cubes is above 1	No
Aydemir and Gunduz (2020)	Fermatean fuzzy TOPSIS method	It can deal with Fermatean fuzzy information with TOPSIS method	Its limitations are similar to FFSs	No
Molodtsov (1999)	Soft sets	It has ability to describe data by multiple attributes	It cannot deal with fuzzy information and multiple experts opinions	No
Alkhazaleh and Salleh (2011)	Soft expert sets	It can capture the evaluations of multiple experts	It cannot deal with fuzzy evaluations	Yes
Alkhazaleh and Salleh (2014)	Fuzzy soft expert sets	It can tackle evaluations of multiple experts in fuzzy environment	It only considers the membership function	Yes
Akram et al. (2021)	*m*-Polar fuzzy soft expert sets	It has ability to deal with multiple experts opinions under *m*-polar fuzzy information	It cannot consider non-membership function	Yes
Broumi and Smarandache (2015)	Intuitionistic fuzzy soft expert sets	It can deal with multiple experts opinions in an intuitionistic fuzzy environment	Its limitations are similar to IFSs	Yes

To summarize, some factors motivating this article are as follows:

1. The FFS model has become a useful and remarkable generalized version of intuitionistic and Pythagorean fuzzy sets, because these existing models rule out situations where the cubic sum of membership and non-membership parts exceeds 1 at some instance.
2. The selection of a best object from a set alternatives in a MCGDM environment is hampered when vague data are forced to adopt the limited form of intuitionistic fuzzy and Pythagorean fuzzy numbers. The aforementioned situations would produce a mutilation of data. A more generalized hybrid model is required in order to ensure telling solutions in such critical situations.
3. FFSESs yield more accurate and precise results when used to cope practical MCGDM problems involving Fermatean fuzzy information as they are an efficient extension of Fermatean fuzzy soft sets regarding experts.
4. The major limitations of the existing intuitionistic and Pythagorean fuzzy hybrid models are two: the restriction of data evaluations to only one expert, and the fact that they cannot deal with data containing Fermatean fuzzy numbers.

The main contributions of this research article are as follows:

1. Soft expert set theory is extended to FFSESs. A detailed discussion analyzes the important properties and preeminent features of the FFSES model.
2. We think out two more efficient algorithms that act in a Fermatean fuzzy environment to deal with practical MCGDM problems. To check its validity, it is applied to a daily life application, namely, the selection of an appropriate solar panel.
3. The accuracy and efficiency of this model is verified with the aid of a comparative analysis with some existing models.

The rest of this article is structured as follows. Section 2 reviews some basic notions, including Fermatean fuzzy sets. Section 3 proposes a new hybrid model called FFSESs, a Fermatean fuzzy extension of SES model, and studies some fundamental properties and operations which are: Fermatean fuzzy soft expert subset, agree-FFSES, disagree-FFSES, equal FFSES, null FFSES, absolute FFSES, AND operation and OR operation. Section 4 describes an application of our proposed MCGDM method, that is, the selection of an appropriate solar technology brand. For that purpose this section provides two algorithms based on FFSESs. Section 5 performs a comparison of the developed model with some existing methods. Section 6 presents the concluding remarks and discusses future research directions.

## Preliminaries

In this section, we first review some basic notions, including Fermatean fuzzy sets (FFSs), score function and accuracy function of FFSs, and soft expert sets (SESs). For other terminologies not discussed in our article, the readers can consult related references (Ali et al. 2019; Bashir and Salleh 2012; Broumi and Smarandache 2015; Ali and Akram 2020; Feng et al. 2020; Zhu and Xu 2018; Feng et al. 2021).

### **Definition 1**

(Senapati and Yager 2020) Let *P* be a universal set. Then a Fermatean fuzzy set is defined by

$$\begin{aligned} A=\{<p,\mu (p),\nu (p)> : p\in P\} \end{aligned}$$

where the functions $\mu ,\nu :P\rightarrow [0,1]$ define respectively the degrees of membership and non-membership of the object $p\in P$ to the set *A*, subject to the following condition for all $p\in P$:

$$\begin{aligned} 0\le (\mu (p))^{3}+(\nu (p))^{3}\le 1. \end{aligned}$$

We denote the collection of all FFSs over *P* by *FFS*(*P*).

The next two definitions allow us to compare FFNs:

### **Definition 2**

(Senapati and Yager 2020) Let ${\mathfrak {F}}=(\mu (p),\nu (p))$ be a FFN over a universe *P*. The score function of ${\mathfrak {F}}$ is defined as

1 $$\begin{aligned} {s({\mathfrak {F}})=\big (\mu (p)\big )^3-\big (\nu (p)\big )^3,} \end{aligned}$$

for all $$p\in P$$.

### **Definition 3**

(Senapati and Yager 2020) Let ${\mathfrak {F}}=(\mu (p),\nu (p))$ be a FFN over a universe *P*. The accuracy function of ${\mathfrak {F}}$ is defined as

2 $$\begin{aligned} {h({\mathfrak {F}})=\big (\mu (p)\big )^3+\big (\nu (p)\big )^3,} \end{aligned}$$

for all $p\in P$. Clearly, $s({\mathfrak {F}}),~h({\mathfrak {F}})\in [0,1].$

### **Definition 4**

(Senapati and Yager 2020) Let ${\mathfrak {F}}_1=(\mu _{1}(p),\nu _{1}(p))$ and ${\mathfrak {F}}_2=(\mu _{2}(p),\nu _{2}(p))$ be any two FFNs over a universe *P*, $s({\mathfrak {F}}_1)$ and $s({\mathfrak {F}}_2)$ be their score functions, and $h({\mathfrak {F}}_1)$ and $h({\mathfrak {F}}_2)$ be their accuracy functions, then

1. if $$s({\mathfrak {F}}_1)> s({\mathfrak {F}}_2)$$ then $${\mathfrak {F}}_1>{\mathfrak {F}}_2$$,
2. if $$s({\mathfrak {F}}_1)=s({\mathfrak {F}}_2)$$ and
  if $$h({\mathfrak {F}}_1)> h({\mathfrak {F}}_2)$$ then $${\mathfrak {F}}_1>{\mathfrak {F}}_2$$,
  if $$h({\mathfrak {F}}_1)= h({\mathfrak {F}}_2)$$ then $${\mathfrak {F}}_1={\mathfrak {F}}_2$$,

### **Definition 5**

(Alkhazaleh and Salleh 2011) Let *P* be a universal set, *E* a universe of parameters, and *Q* a set of experts. Let $O=\{0=\mathrm{disagree}, 1=\mathrm{agree}\}$ be the set of their opinions. For any $A \subseteq X$, a pair (*F*, *A*) is said to be a soft expert set over *P*, where $X = E \times Q \times O$ and *F* is a function given by

$$\begin{aligned} F:A\rightarrow 2^P, \end{aligned}$$

where $2^P$ denotes the collection of all subsets of *P*.

## Fermatean fuzzy soft expert sets

In this section, we present the main concept of this study, that is, the FFSES model, along with a few of its useful properties.

### **Definition 6**

Let *P* be a universal set, *E* a universe of parameters, and *Q* a set of experts. Let $O=\{0=\mathrm{disagree}, 1=\mathrm{agree}\}$ be the collection of opinions of experts. For any $A \subseteq Y$, a triplet $(\lambda ,A)$ is referred to as a *Fermatean fuzzy soft expert set* or FFSES where $Y = E\times Q \times O$ and $\lambda$ is a mapping given below:

$$\begin{aligned} \lambda :A\rightarrow FFS(P) \end{aligned}$$

which satisfy the condition that for all $\epsilon \in A,~{q\in Q}$ there exists exactly one $(q,p_\epsilon )\in Q\times P$ such that $(q,p_\epsilon )\in \lambda (\epsilon )$, i.e., for all $\epsilon \in A, q \in Q$, and $p\in P$, we get

$$\begin{aligned} (\lambda ,A)=\{\langle \epsilon , \big (p,\mu (p),\nu (p)\big )\rangle :\epsilon \in A, ~p\in P\}, \end{aligned}$$

with the following condition:

$$\begin{aligned} 0\le (\mu (p))^{3}+(\nu (p))^{3}\le 1. \end{aligned}$$

Another compact and precise way to represent a FFSES $(\lambda ,{A})$ is its tabular representation. To describe it, assume that $P=\{p_1,p_2,\ldots ,p_n\}$ is a universal set, and $E=\{e_1,e_2,\ldots ,e_m\}$ is a universe of parameters about the elements of *P*. Let $Q=\{q_1,q_2,\ldots ,q_t\}$ be a set of experts and $O=\{0= {\mathrm{disagree}}, 1= {\mathrm{agree}}\}$ be their opinions. Then, a FFSES $(\lambda ,{A})$ can be presented by an alternative tabular arrangement as shown in Table 2 below:

**Table 2:** Tabular arrangement of the FFSES $(\lambda ,A)$

$(\lambda ,A)$	$p_1$	$\ldots$	$p_n$
$({e}_1, {q}_1, 1)$	$\big (\mu _{({e}_1, {q}_1, 1)}(p_1),\nu _{({e}_1, {q}_1, 1)}(p_1)\big )$	$\ldots$	$\big (\mu _{({e}_1, {q}_1, 1)}(p_n),\nu _{({e}_1, {q}_1, 1)}(p_n)\big )$
$({e}_1, {q}_2, 1)$	$\big (\mu _{({e}_1, {q}_2, 1)}(p_1),\nu _{({e}_1, {q}_2, 1)}(p_1)\big )$	$\ldots$	$\big (\mu _{({e}_1, {q}_2, 1)}(p_n),\nu _{({e}_1, {q}_2, 1)}(p_n)\big )$
$\vdots$	$\vdots$	$\vdots$	$\vdots$
$({e}_m, {q}_t, 1)$	$\big (\mu _{({e}_m, {q}_t, 1)}(p_1),\nu _{({e}_m, {q}_t, 1)}(p_1)\big )$	$\ldots$	$\big (\mu _{({e}_m, {q}_t, 1)}(p_n),\nu _{({e}_m, {q}_t, 1)}(p_n)\big )$
$({e}_1, {q}_1, 0)$	$\big (\mu _{({e}_1, {q}_1, 0)}(p_1),\nu _{({e}_1, {q}_1, 0)}(p_1)\big )$	$\ldots$	$\big (\mu _{({e}_1, {q}_1, 0)}(p_n),\nu _{({e}_1, {q}_1, 0)}(p_n)\big )$
$({e}_1, {q}_2, 0)$	$\big (\mu _{({e}_1, {q}_2, 0)}(p_1),\nu _{({e}_1, {q}_2, 0)}(p_1)\big )$	$\ldots$	$\big (\mu _{({e}_1, {q}_2, 0)}(p_n),\nu _{({e}_1, {q}_2, 0)}(p_n)\big )$
$\vdots$	$\vdots$	$\vdots$	$\vdots$
$({e}_m, {q}_t, 0)$	$\big (\mu _{({e}_m, {q}_t, 0)}(p_1),\nu _{({e}_m, {q}_t, 0)}(p_1)\big )$	$\ldots$	$\big (\mu _{({e}_m, {q}_t, 0)}(p_n),\nu _{({e}_m, {q}_t, 0)}(p_n)\big )$

This novel notion is explained by an example provided below:

### *Example 1*

Suppose a largest hotel company XYZ wants to buy a hotel from the five options $p_1,p_2,p_3,p_4$ and $p_5$. To choose an appropriate option according to the need of company, they decide to take opinions and judgments of three experts having expertise in the field. Let $P=\{p_1,p_2,\ldots ,p_5\}$ be a set of hotels and $Q=\{q_1,q_2,q_3\}$ be the set of three experts, $E=\{e_1=\mathrm{location},e_2=\mathrm{design}, e_3=\mathrm{technology},e_4=\mathrm{entrance}\}$ a set of parameters which have important role in the selection of hotel, and $Y=E\times Q\times O$.

Then all the information of experts opinions about the alternatives is given in the form of a FFSES $(\lambda ,A)$, $A\subseteq Y$ as the collection of approximations provided below:

$$\begin{aligned} (\lambda ,A)=\big \{&\big ((e_1,q_1,1),\{(p_1,0.5,0.7),(p_2,0.8,0.4), (p_3,0.7,0.6),(p_4,0.4,0.8),(p_5,0.6,0.4)\}\big ),\\&\big ((e_1,q_2,1),\{(q_1,0.9,0.3),(p_2,0.4,0.9),(p_3,0.5,0.7), (p_4,o.4,0.7),(p_5,0.7,0.5)\}\big ),\\ {}&\big ((e_1,q_3,1),\{(p_1,0.8,0.7), (p_2,0.4,0.8),(p_3,0.7,0.6),(p_4,0.4,0.6),(p_5,0.4,0.9)\}\big ),\\&\big ((e_2,q_1,1),\{(p_1,0.5,0.7),(p_2,0.7,0.4),(p_3,0.8,0.4), (p_4,0.4,0.6),(p_5,0.6,0.7)\}\big ),\\&\big ((e_2,q_2,1),\{(p_1,0.6,0.5),(p_2,0.4,0.6),(p_3,0.8,0.4), (p_4,0.5,0.4),(p_5,0.9,0.2)\}\big ),\\ {}&\big ((e_2,q_3,1),\{(p_1,0.5,0.7), (p_2,0.8,0.4),(p_3,0.6,0.5),(p_4,0.4,0.6),(p_5,0.8,0.7)\}\big ),\\&\big ((e_3,q_1,1),\{(p_1,0.8,0.4),(p_2,0.9,0.4),(p_3,0.7,0.6), (p_4,0.8,0.4),(p_5,0.4,0.6)\}\big ),\\&\big ((e_3,q_2,1),\{(p_1,0.8,0.4), (p_2,0.9,0.4),(p_3,0.7,0.6),(p_4,0.6,0.4),(p_5,0.2,0.8)\}\big ),\\&\big ((e_3,q_2,1),\{(p_1,0.5,0.6),(p_2,0.9,0.4),(p_3,0.7,0.6), (p_4,0.8,0.4),(p_5,0.4,0.6)\}\big ),\\ {}&\big ((e_3,q_3,1),\{(p_1,0.8,0.4), (p_2,0.4,0.5),(p_3,0.7,0.5),(p_4,0.5,0.6),(p_5,0.6,0.7)\}\big ),\\&\big ((e_4,q_1,1),\{(p_1,0.5,0.6),(p_2,0.8,0.7),(p_3,0.4,0.9), (p_4,0.4,0.5),(p_5,0.7,0.3)\}\big ),\\ {}&\big ((e_4,q_2,1),\{(p_1,0.2,0.9), (p_2,0.8,0.2),(p_3,0.6,0.5),(p_4,0.7,0.3),(p_5,0.4,0.6)\}\big ),\\&\big ((e_4,q_3,1),\{(p_1,0.8,0.4),(p_2,0.6,0.4),(p_3,0.8,0.3), (p_4,0.4,0.7),(p_5,0.9,0.2)\}\big ),\\ {}&\big ((e_1,q_1,0),\{(p_1,0.5,0.9), (p_2,0.8,0.6),(p_3,0.9,0.2),(p_4,0.5,0.5),(p_5,0.9,0.1)\}\big ),\\&\big ((e_1,q_2,0),\{(p_1,0.6,0.5),(p_2,0.8,0.4),(p_3,0.5,0.4), (p_4,0.9,0.3),(p_5,0.5,0.4)\}\big ),\\ {}&\big ((e_1,q_3,0),\{(p_1,0.8,0.7), (p_2,0.9,0.4),(p_3,0.7,0.8),(p_4,0.5,0.7),(p_5,0.4,0.7)\}\big ),\\&\big ((e_2,q_1,0),\{(p_1,0.6,0.8),(p_2,0.5,0.8),(p_3,0.9,0.3),(p_4,0.8,0.4), (p_5,0.7,0.6)\}\big ),\\ {}&\big ((e_2,q_2,0),\{(p_1,0.8,0.7),(p_2,0.7,0.6), (p_3,0.5,0.3),(p_4,0.7,0.6),(p_5,0.9,0.2)\}\big ),\\&\big ((e_2,q_3,0), \{(p_1,0.5,0.8),(p_2,0.9,0.5),(p_3,0.8,0.6),(p_4,0.9,0.6),(p_5,0.8,0.7)\} \big ),\\&\big ((e_3,q_1,0),\{(p_1,0.5,0.7),(p_2,0.7,0.8), (p_3,0.5,0.4),(p_4,0.9,0.1),(p_5,0.7,0.6)\}\big ),\\&\big ((e_3,q_2,0),\{(p_1,0.9,0.3),(p_2,0.5,0.4),(p_3,0.6,0.8), (p_4,0.5,0.6),(p_5,0.9,0.3)\}\big ),\\&\big ((e_3,q_3,0), \{(p_1,0.9,0.1),(p_2,0.6,0.6),(p_3,0.9,0.4),(p_4,0.8,0.4),(p_5,0.2,0.9)\} \big ),\\&\big ((e_4,q_1,0),\{(p_1,0.5,0.4),(p_2,0.8,0.5),(p_3,0.7,0.2), (p_4,0.5,0.6),(p_5,0.8,0.3)\}\big ),\\&\big ((e_4,q_2,0),\{(p_1,0.8,0.4),(p_2,0.9,0.3), (p_3,0.6,0.2),(p_4,0.7,0.5),(p_5,0.5,0.8)\}\big ),\\&\big ((e_4,q_3,0),\{(p_1,0.7,0.6),(p_2,0.8,0.3),(p_3,0.5,0.8), (p_4,0.5,0.7),(p_5,0.8,0.1)\}\big ) \} \end{aligned}$$

Note that according to the expert ‘$q_1$’ hotel $p_1$ is suitable with membership score 0.5 and non-membership score 0.7 regarding parameter location $e_1$. Similarly, the expert ‘$q_1$’ agrees that the hotel $p_2$ is suitable with membership score 0.8 and non-membership score 0.4 regarding parameter location $e_1$ and so on. The tabular representation of FFSES is given in Table 3.

**Table 3:** Tabular representation of the FFSES $(\lambda ,A)$

$(\lambda ,A)$	$p_1$	$p_2$	$p_3$	$p_4$	$p_5$
$(e_1,q_1,1)$	(0, 5, 0.7)	(0.8, 0.4)	(0.7, 0.6)	(0.4, 0.8)	(0.6, 0.4)
$(e_1,q_2,1)$	(0.9, 0.3)	(0.4, 0.9)	(0.5, 0.7)	(0.4, 0.7)	(0.7, 0.5)
$(e_1,q_3,1)$	(0.8, 0.7)	(0.4, 0.8)	(0.7, 0.6)	(0.4, 0.6)	(0.4, 0.9)
$(e_2,q_1,1)$	(0.5, 0.7)	(0.7, 0.4)	(0.8, 0.4)	(0.4, 0.6)	(0.6, 0.7)
$(e_2,q_2,1)$	(0.6, 0.5)	(0.4, 0.6)	(0.8, 0.4)	(0.5, 0.4)	(0.9, 0.2)
$(e_2,q_3,1)$	(0.5, 0.7)	(0.8, 0.4)	(0.6, 0.5)	(0.4, 0.6)	(0.8, 0.7)
$(e_3,q_1,1)$	(0.8, 0.4)	(0.9, 0.4)	(0.7, 0.6)	(0.8, 0.4)	(0.4, 0.6)
$(e_3,q_2,1)$	(0.7, 0.5)	(0.6, 0.6)	(0.8, 0.5)	(0.7, 0.5)	(0.3, 0.6)
$(e_3,q_3,1)$	(0.8, 0.4)	(0.4, 0.5)	(0.7, 0.5)	(0.5, 0.6)	(0.6, 0.7)
$(e_4,q_1,1)$	(0.5, 0.6)	(0.8, 0.7)	(0.4, 0.9)	(0.4, 0.5)	(0.7, 0.3)
$(e_4,q_2,1)$	(0.2, 0.9)	(0.8, 0.2)	(0.6, 0.5)	(0.7, 0.3)	(0.4, 0.6)
$(e_4,q_3,1)$	(0.8, 0.4)	(0.6, 0.4)	(0.8, 0.3)	(0.4, 0.7)	(0.9, 0.2)
$(e_1,q_1,0)$	(0.5, 0.9)	(0.8, 0.6)	(0.9, 0.2)	(0.5, 0.5)	(0.9, 0.1)
$(e_1,q_2,0)$	(0.6, 0.5)	(0.8, 0.4)	(0.5, 0.4)	(0.9, 0.3)	(0.5, 0.4)
$(e_1,q_3,0)$	(0.8, 0.7)	(0.9, 0.4)	(0.7, 0.8)	(0.5, 0.7)	(0.4, 0.7)
$(e_2,q_1,0)$	(0.6, 0.8)	(0.5, 0.8)	(0.9, 0.3)	(0.8, 0.4)	(0.7, 0.6)
$(e_2,q_2,0)$	(0.8, 0.7)	(0.7, 0.6)	(0.5, 0.3)	(0.7, 0.6)	(0.9, 0.2)
$(e_2,q_3,0)$	(0.5, 0.8)	(0.9, 0.5)	(0.8, 0.6)	(0.9, 0.6)	(0.8, 0.7)
$(e_3,q_1,0)$	(0.5, 0.7)	(0.7, 0.8)	(0.5, 0.4)	(0.9, 0.1)	(0.7, 0.6)
$(e_3,q_2,0)$	(0.9, 0.3)	(0.5, 0.4)	(0.6, 0.8)	(0.5, 0.6)	(0.9, 0.3)
$(e_3,q_3,0)$	(0.9, 0.1)	(0.6, 0.6)	(0.9, 0.4)	(0.8, 0.4)	(0.2, 0.9)
$(e_4,q_1,0)$	(0.5, 0.4)	(0.8, 0.5)	(0.7, 0.2)	(0.5, 0.6)	(0.8, 0.3)
$(e_4,q_2,0)$	(0.8, 0.4)	(0.9, 0.3)	(0.6, 0.2)	(0.7, 0.5)	(0.5, 0.8)
$(e_4,q_3,0)$	(0.7, 0.6)	(0.8, 0.3)	(0.5, 0.8)	(0.5, 0.7)	(0.8, 0.1)

Another useful concept, namely, Fermatean fuzzy soft expert subset is discussed as follows:

### **Definition 7**

Let $(\lambda ,A)$ and $(\zeta ,B)$ be two FFSESs on the universe *P*. Then $(\lambda ,A)$ is said to be a Fermatean fuzzy soft expert subset of $(\zeta ,B)$ if

1. $$A\subseteq B,$$
2. $~\lambda (\epsilon )$ is a Fermatean fuzzy subset of $\zeta (\epsilon )$, (i.e., $\forall ~\epsilon \in A,~\lambda (\epsilon )\subseteq \zeta (\epsilon )$) .

It is denoted by $(\lambda ,A){\hat{\subseteq }}(\zeta ,B)$. Note that $(\zeta ,B)$ is said to be a Fermatean fuzzy soft expert superset of $(\lambda ,A)$.

The following example explains this novel concept:

### *Example 2*

Consider Example 1. Let $(\lambda ,A)$ and $(\zeta ,B)$ be two FFSESs over *P* where

$$\begin{aligned} A&=\{(e_1,q_1,1),(e_1,q_2,1),(e_1,q_3,1),(e_3,q_1,0),(e_3,q_2,1), (e_3,q_3,0)\},\\ B&=\{(e_1,q_1,1),(e_1,q_2,1),(e_1,q_3,1),(e_3,q_1,0),(e_3,q_2,1), (e_3,q_3,0)\} \end{aligned}$$

are displayed by Tables 4 and 5, respectively. It is clear that $A\subseteq B$.

**Table 4:** Tabular representation of the FFSES $(\lambda ,A)$

$(\lambda ,A)$	$p_1$	$p_2$	$p_3$	$p_4$	$p_5$
$(e_1,q_1,1)$	(0, 4, 0.7)	(0.7, 0.4)	(0.6, 0.6)	(0.4, 0.8)	(0.5, 0.4)
$(e_1,q_2,1)$	(0.8, 0.3)	(0.4, 0.9)	(0.5, 0.7)	(0.4, 0.7)	(0.6, 0.5)
$(e_1,q_3,1)$	(0.6, 0.7)	(0.4, 0.8)	(0.6, 0.6)	(0.4, 0.6)	(0.4, 0.9)
$(e_3,q_1,0)$	(0.5, 0.7)	(0.6, 0.8)	(0.5, 0.4)	(0.8, 0.1)	(0.6, 0.6)
$(e_3,q_2,1)$	(0.6, 0.5)	(0.6, 0.6)	(0.7, 0.5)	(0.6, 0.5)	(0.3, 0.6)
$(e_3,q_3,0)$	(0.8, 0.1)	(0.6, 0.6)	(0.7, 0.4)	(0.7, 0.4)	(0.2, 0.9)

**Table 5:** Tabular representation of the FFSES $(\zeta ,B)$

$(\zeta ,B)$	$p_1$	$p_2$	$p_3$	$p_4$	$p_5$
$(e_1,q_1,1)$	(0, 5, 0.6)	(0.8, 0.4)	(0.7, 0.5)	(0.4, 0.6)	(0.6, 0.4)
$(e_1,q_2,1)$	(0.9, 0.3)	(0.4, 0.7)	(0.5, 0.6)	(0.4, 0.6)	(0.7, 0.5)
$(e_1,q_3,1)$	(0.8, 0.6)	(0.4, 0.7)	(0.7, 0.6)	(0.4, 0.6)	(0.4, 0.7)
$(e_3,q_1,0)$	(0.5, 0.6)	(0.7, 0.6)	(0.5, 0.4)	(0.9, 0.1)	(0.7, 0.5)
$(e_3,q_2,1)$	(0.7, 0.5)	(0.6, 0.6)	(0.8, 0.5)	(0.7, 0.5)	(0.3, 0.6)
$(e_3,q_3,0)$	(0.9, 0.1)	(0.6, 0.6)	(0.9, 0.4)	(0.8, 0.4)	(0.2, 0.8)

Clearly, $$(\lambda ,A){\hat{\subseteq }}(\zeta ,B)$$.

Now we give two concepts, namely, agree- and disagree-FFSESs, and illustrate them via examples:

### **Definition 8**

Let $(\lambda ,A)$ be a FFSESs on the universe *P*. Then its agree-FFSESs $(\lambda ,A)_1$ on *P* is a Fermatean fuzzy soft expert subset of $(\lambda ,A)$ which is given by

$$\begin{aligned} (\lambda ,A)_1=\{\lambda (\epsilon ):\epsilon \in E\times Q\times \{1\}\} \end{aligned}$$

### *Example 3*

Reconsider FFSES as given in Example 1. Then, its agree-FFSES $(\lambda ,A)_1$ can be viewed in Table 6.

**Table 6:** Tabular arrangement of the agree-FFSES $(\lambda ,A)_1$

$(\lambda ,A)_1$	$p_1$	$p_2$	$p_3$	$p_4$	$p_5$
$(e_1,q_1,1)$	(0, 5, 0.7)	(0.8, 0.4)	(0.7, 0.6)	(0.4, 0.8)	(0.6, 0.4)
$(e_1,q_2,1)$	(0.9, 0.3)	(0.4, 0.9)	(0.5, 0.7)	(0.4, 0.7)	(0.7, 0.5)
$(e_1,q_3,1)$	(0.8, 0.7)	(0.4, 0.8)	(0.7, 0.6)	(0.4, 0.6)	(0.4, 0.9)
$(e_2,q_1,1)$	(0.5, 0.7)	(0.7, 0.4)	(0.8, 0.4)	(0.4, 0.6)	(0.6, 0.7)
$(e_2,q_2,1)$	(0.6, 0.5)	(0.4, 0.6)	(0.8, 0.4)	(0.5, 0.4)	(0.9, 0.2)
$(e_2,q_3,1)$	(0.5, 0.7)	(0.8, 0.4)	(0.6, 0.5)	(0.4, 0.6)	(0.8, 0.7)
$(e_3,q_1,1)$	(0.8, 0.4)	(0.9, 0.4)	(0.7, 0.6)	(0.8, 0.4)	(0.4, 0.6)
$(e_3,q_2,1)$	(0.7, 0.5)	(0.6, 0.6)	(0.8, 0.5)	(0.7, 0.5)	(0.3, 0.6)
$(e_3,q_3,1)$	(0.8, 0.4)	(0.4, 0.5)	(0.7, 0.5)	(0.5, 0.6)	(0.6, 0.7)
$(e_4,q_1,1)$	(0.5, 0.6)	(0.8, 0.7)	(0.4, 0.9)	(0.4, 0.5)	(0.7, 0.3)
$(e_4,q_2,1)$	(0.2, 0.9)	(0.8, 0.2)	(0.6, 0.5)	(0.7, 0.3)	(0.4, 0.6)
$(e_4,q_3,1)$	(0.8, 0.4)	(0.6, 0.4)	(0.8, 0.3)	(0.4, 0.7)	(0.9, 0.2)

### **Definition 9**

Let $(\lambda ,A)$ be a FFSES on the universe *P*. Then its disagree-FFSES $(\lambda ,A)_0$ on *P* is a Fermatean fuzzy soft expert subset of $(\lambda ,A)$ which is given by

$$\begin{aligned} (\lambda ,A)_0=\{\lambda (\epsilon ):\epsilon \in E\times Q\times \{0\}\}. \end{aligned}$$

### *Example 4*

Reconsider FFSES as given in Example 1. Then, its disagree-FFSES $(\lambda ,A)_0$ can be seen in Table 7.

**Table 7:** Tabular arrangement of the disagree-FFSESs $(\lambda ,A)_0$

$(\lambda ,A)_0$	$p_1$	$p_2$	$p_3$	$p_4$	$p_5$
$(e_1,q_1,0)$	(0.5, 0.9)	(0.8, 0.6)	(0.9, 0.2)	(0.5, 0.5)	(0.9, 0.1)
$(e_1,q_2,0)$	(0.6, 0.5)	(0.8, 0.4)	(0.5, 0.4)	(0.9, 0.3)	(0.5, 0.4)
$(e_1,q_3,0)$	(0.8, 0.7)	(0.9, 0.4)	(0.7, 0.8)	(0.5, 0.7)	(0.4, 0.7)
$(e_2,q_1,0)$	(0.6, 0.8)	(0.5, 0.8)	(0.9, 0.3)	(0.8, 0.4)	(0.7, 0.6)
$(e_2,q_2,0)$	(0.8, 0.7)	(0.7, 0.6)	(0.5, 0.3)	(0.7, 0.6)	(0.9, 0.2)
$(e_2,q_3,0)$	(0.5, 0.8)	(0.9, 0.5)	(0.8, 0.6)	(0.9, 0.6)	(0.8, 0.7)
$(e_3,q_1,0)$	(0.5, 0.7)	(0.7, 0.8)	(0.5, 0.4)	(0.9, 0.1)	(0.7, 0.6)
$(e_3,q_2,0)$	(0.9, 0.3)	(0.5, 0.4)	(0.6, 0.8)	(0.5, 0.6)	(0.9, 0.3)
$(e_3,q_3,0)$	(0.9, 0.1)	(0.6, 0.6)	(0.9, 0.4)	(0.8, 0.4)	(0.2, 0.9)
$(e_4,q_1,0)$	(0.5, 0.4)	(0.8, 0.5)	(0.7, 0.2)	(0.5, 0.6)	(0.8, 0.3)
$(e_4,q_2,0)$	(0.8, 0.4)	(0.9, 0.3)	(0.6, 0.2)	(0.7, 0.5)	(0.5, 0.8)
$(e_4,q_3,0)$	(0.7, 0.6)	(0.8, 0.3)	(0.5, 0.8)	(0.5, 0.7)	(0.8, 0.1)

We now provide some basic notions for FFSESs, namely, equality between FFSESs, null and absolute FFSESs.

### **Definition 10**

Let $(\lambda ,A)$ and $(\zeta ,B)$ be two FFSESs on the universe *P*. Then $(\lambda ,A)$ and $(\zeta ,B)$ are said to be equal FFSESs if $(\lambda ,A)$ is a Fermatean fuzzy soft expert subset of $(\zeta ,B)$ and $(\zeta ,B)$ is a Fermatean fuzzy soft expert subset of $(\lambda ,A)$.

### **Definition 11**

A FFSES $(\lambda ,A)$ is said to be a null FFSES, denoted by $(\phi ,A)$ and is defined as below:

$$\begin{aligned} (\phi ,A)=\lambda (\alpha )=\langle (0,1)\rangle \end{aligned}$$

for all $$\alpha \in A$$.

### **Definition 12**

A FFSES $(\lambda ,A)$ is said to be an absolute FFSES, denoted by $(\Omega ,A)$ and is defined by

$$\begin{aligned} (\Omega ,A)=\lambda (\alpha )=\langle (1,0)\rangle \end{aligned}$$

for all $$\alpha \in A$$.

Now we investigate some basic operations and properties of FFSESs, namely, complement, intersection, union, AND operation and OR operation with numerical examples. We start with complement of FFSESs.

### **Definition 13**

Let $(\lambda ,A)$ be a FFSES on the universe *P*. Then its complement is represented by $(\lambda ,A)^c$ and is given as $(\lambda ,A)^c=(\lambda ^c,\sim A)$ where $\lambda ^c:\sim A\rightarrow FFS(P)$ is a function given by $\lambda ^c(\lnot \epsilon )=(\nu _\epsilon (p),\mu _\epsilon (p))$ such as $(\mu _\epsilon (p),\nu _\epsilon (p))\in \lambda (\epsilon )$ for all $\epsilon \in A,~p\in P.$

### *Example 5*

Consider FFSES $(\lambda , A)$ in Example 1. Then, its complement $(\lambda , A)^c$ is given by Table 8.

**Table 8:** Tabular representation of complement of the FFSES $(\lambda ,A)^c$

$(\lambda ,A)^c$	$p_1$	$p_2$	$p_3$	$p_4$	$p_5$
$(\lnot e_1,q_1,1)$	(0.7, 0.5)	(0.4, 0.8)	(0.6, 0.7)	(0.8, 0.4)	(0.4, 0.6)
$(\lnot e_1,q_2,1)$	(0.3, 0.9)	(0.9, 0.4)	(0.7, 0.5)	(0.7, 0.4)	(0.5, 0.7)
$(\lnot e_1,q_3,1)$	(0.7, 0.8)	(0.8, 0.4)	(0.6, 0.7)	(0.6, 0.4)	(0.9, 0.4)
$(\lnot e_2,q_1,1)$	(0.7, 0.5)	(0.4, 0.7)	(0.4, 0.8)	(0.6, 0.4)	(0.7, 0.6)
$(\lnot e_2,q_2,1)$	(0.5, 0.6)	(0.6, 0.4)	(0.4, 0.8)	(0.4, 0.5)	(0.2, 0.9)
$(\lnot e_2,q_3,1)$	(0.7, 0.5)	(0.4, 0.8)	(0.5, 0.6)	(0.6, 0.4)	(0.7, 0.8)
$(\lnot e_3,q_1,1)$	(0.4, 0.8)	(0.4, 0.9)	(0.6, 0.7)	(0.4, 0.8)	(0.6, 0.4)
$(\lnot e_3,q_2,1)$	(0.5, 0.7)	(0.6, 0.6)	(0.5, 0.8)	(0.5, 0.7)	(0.6, 0.3)
$(\lnot e_3,q_3,1)$	(0.4, 0.8)	(0.5, 0.4)	(0.5, 0.7)	(0.6, 0.5)	(0.7, 0.6)
$(\lnot e_4,q_1,1)$	(0.6, 0.5)	(0.7, 0.8)	(0.9, 0.4)	(0.5, 0.4)	(0.3, 0.7)
$(\lnot e_4,q_2,1)$	(0.9, 0.2)	(0.2, 0.8)	(0.5, 0.6)	(0.3, 0.7)	(0.6, 0.4)
$(\lnot e_4,q_3,1)$	(0.4, 0.8)	(0.4, 0.6)	(0.3, 0.8)	(0.7, 0.4)	(0.2, 0.9)
$(\lnot e_1,q_1,0)$	(0.9, 0.5)	(0.6, 0.8)	(0.2, 0.9)	(0.5, 0.5)	(0.1, 0.9)
$(\lnot e_1,q_2,0)$	(0.5, 0.6)	(0.4, 0.8)	(0.4, 0.5)	(0.3, 0.9)	(0.4, 0.5)
$(\lnot e_1,q_3,0)$	(0.7, 0.8)	(0.4, 0.9)	(0.8, 0.7)	(0.7, 0.5)	(0.7, 0.4)
$(\lnot e_2,q_1,0)$	(0.8, 0.6)	(0.8, 0.5)	(0.3, 0.9)	(0.4, 0.8)	(0.6, 0.7)
$(\lnot e_2,q_2,0)$	(0.7, 0.8)	(0.6, 0.7)	(0.3, 0.5)	(0.6, 0.7)	(0.2, 0.9)
$(\lnot e_2,q_3,0)$	(0.8, 0.5)	(0.5, 0.9)	(0.6, 0.8)	(0.6, 0.9)	(0.7, 0.8)
$(\lnot e_3,q_1,0)$	(0.7, 0.5)	(0.8, 0.7)	(0.4, 0.5)	(0.1, 0.9)	(0.6, 0.7)
$(\lnot e_3,q_2,0)$	(0.3, 0.9)	(0.4, 0.5)	(0.8, 0.6)	(0.6, 0.5)	(0.3, 0.9)
$(\lnot e_3,q_3,0)$	(0.1, 0.9)	(0.6, 0.6)	(0.4, 0.9)	(0.4, 0.8)	(0.9, 0.2)
$(\lnot e_4,q_1,0)$	(0.4, 0.5)	(0.5, 0.8)	(0.2, 0.7)	(0.6, 0.5)	(0.3, 0.8)
$(\lnot e_4,q_2,0)$	(0.4, 0.8)	(0.3, 0.9)	(0.2, 0.6)	(0.5, 0.7)	(0.8, 0.5)
$(\lnot e_4,q_3,0)$	(0.6, 0.7)	(0.3, 0.8)	(0.8, 0.5)	(0.7, 0.5)	(0.1, 0.8)

The following proposition explains an important property related to complement of FFSESs.

### **Proposition 1**

*Let* $(\lambda ,A)$ *be a FFSES on the universe P. Then*

1. $$((\lambda ,A)^c)^c=(\lambda ,A)$$,

### *Proof*

Straightforward. $$\square$$

Now we define some more useful notions of FFSESs, which are: union, intersection, AND and OR operations of FFSESs, respectively, and we explain them by corresponding examples.

### **Definition 14**

Let $(\lambda ,A)$ and $(\zeta ,B)$ be two FFSESs on the universe *P*. Then, we define their union by $(H,K)=(\lambda ,A)\Cup (\zeta ,B)$, which is again a FFSES where $K=A\cup B$ and $\forall ~\epsilon \in K$,

$$\begin{aligned} H(\epsilon )= {\left\{ \begin{array}{ll} \lambda (\epsilon ),&\quad{\mathrm{if}}\;\epsilon \in A-B,\\ \zeta (\epsilon ),&\quad{\mathrm{if}}\;\epsilon \in B-A,\\ \lambda (\epsilon )\cup \zeta (\epsilon )&\quad{\mathrm{if}}\;\epsilon \in A\cap B, \end{array}\right. } \end{aligned}$$

where $\lambda (\epsilon )\cup \zeta (\epsilon )=(\max (\alpha ^1_\epsilon , \alpha ^2_\epsilon ),\min (\beta ^1_\epsilon , \beta ^2_\epsilon )),$ for all $(\alpha ^1_\epsilon ,\beta ^1_\epsilon )\in \lambda (\epsilon ),~(\alpha ^2_\epsilon ,\beta ^2_\epsilon )\in \zeta (\epsilon ).$

### *Example 6*

Let $(\lambda ,A)$ and $(\zeta ,B)$ be two FFSESs over the universe $P=\{p_1,p_2,\ldots ,p_5\}$ which are respectively displayed by Tables 9 and 10 where

$$\begin{aligned} A&=\{(e_1,q_1,1),(e_1,q_2,1),(e_1,q_3,1),(e_2,q_1,1),(e_2,q_2,1), (e_2,q_3,1),(e_3,q_1,1),(e_3,q_2,1),\\&qquad (e_3,q_3,1),(e_4,q_1,1), (e_4,q_2,1),(e_4,q_3,1),(e_1,q_1,0),(e_1,q_2,0),(e_1,q_3,0),(e_2,q_1,0),\\&qquad (e_2,q_2,0),(e_2,q_3,0),(e_3,q_1,0),(e_3,q_2,0),(e_3,q_3,0),(e_4,q_1,0) ,(e_4,q_2,0),(e_4,q_3,0)\}\\ B&=\{(e_1,q_1,1),(e_1,q_2,1),(e_1,q_3,1),(e_2,q_1,1),(e_2,q_2,1), (e_2,q_3,1),(e_3,q_1,1),(e_3,q_2,1),\\&qquad (e_3,q_3,1),(e_4,q_1,1), (e_4,q_2,1),(e_4,q_3,1),(e_1,q_1,0),(e_1,q_2,0),(e_1,q_3,0),(e_2,q_1,0),\\&qquad(e_2,q_2,0),(e_2,q_3,0),(e_3,q_1,0),(e_3,q_2,0),(e_3,q_3,0),(e_4,q_1,0), (e_4,q_2,0),(e_4,q_3,0)\} \end{aligned}$$

**Table 9:** Tabular representation of the FFSES $(\lambda ,A)$

$(\lambda ,A)$	$p_1$	$p_2$	$p_3$	$p_4$	$p_5$
$(e_1,q_1,1)$	(0.5, 0.7)	(0.8, 0.4)	(0.7, 0.6)	(0.4, 0.8)	(0.6, 0.4)
$(e_1,q_2,1)$	(0.9, 0.3)	(0.4, 0.9)	(0.5, 0.7)	(0.4, 0.7)	(0.7, 0.5)
$(e_1,q_3,1)$	(0.8, 0.7)	(0.4, 0.8)	(0.7, 0.6)	(0.4, 0.6)	(0.4, 0.9)
$(e_2,q_1,1)$	(0.5, 0.7)	(0.7, 0.4)	(0.8, 0.4)	(0.4, 0.6)	(0.6, 0.7)
$(e_2,q_2,1)$	(0.6, 0.5)	(0.4, 0.6)	(0.8, 0.4)	(0.5, 0.4)	(0.9, 0.2)
$(e_2,q_3,1)$	(0.5, 0.7)	(0.8, 0.4)	(0.6, 0.5)	(0.4, 0.6)	(0.8, 0.7)
$(e_3,q_1,1)$	(0.8, 0.4)	(0.9, 0.4)	(0.7, 0.6)	(0.8, 0.4)	(0.4, 0.6)
$(e_3,q_2,1)$	(0.7, 0.5)	(0.6, 0.6)	(0.8, 0.5)	(0.7, 0.5)	(0.3, 0.6)
$(e_3,q_3,1)$	(0.8, 0.4)	(0.4, 0.5)	(0.7, 0.5)	(0.5, 0.6)	(0.6, 0.7)
$(e_4,q_1,1)$	(0.5, 0.6)	(0.8, 0.7)	(0.4, 0.9)	(0.4, 0.5)	(0.7, 0.3)
$(e_4,q_2,1)$	(0.2, 0.9)	(0.8, 0.2)	(0.6, 0.5)	(0.7, 0.3)	(0.4, 0.6)
$(e_4,q_3,1)$	(0.8, 0.4)	(0.6, 0.4)	(0.8, 0.3)	(0.4, 0.7)	(0.9, 0.2)
$(e_1,q_1,0)$	(0.5, 0.9)	(0.8, 0.6)	(0.9, 0.2)	(0.5, 0.5)	(0.9, 0.1)
$(e_1,q_2,0)$	(0.6, 0.5)	(0.8, 0.4)	(0.5, 0.4)	(0.9, 0.3)	(0.5, 0.4)
$(e_1,q_3,0)$	(0.8, 0.7)	(0.9, 0.4)	(0.7, 0.8)	(0.5, 0.7)	(0.4, 0.7)
$(e_2,q_1,0)$	(0.6, 0.8)	(0.5, 0.8)	(0.9, 0.3)	(0.8, 0.4)	(0.7, 0.6)
$(e_2,q_2,0)$	(0.8, 0.7)	(0.7, 0.6)	(0.5, 0.3)	(0.7, 0.6)	(0.9, 0.2)
$(e_2,q_3,0)$	(0.5, 0.8)	(0.9, 0.5)	(0.8, 0.6)	(0.9, 0.6)	(0.8, 0.7)
$(e_3,q_1,0)$	(0.5, 0.7)	(0.7, 0.8)	(0.5, 0.4)	(0.9, 0.1)	(0.7, 0.6)
$(e_3,q_2,0)$	(0.9, 0.3)	(0.5, 0.4)	(0.6, 0.8)	(0.5, 0.6)	(0.9, 0.3)
$(e_3,q_3,0)$	(0.9, 0.1)	(0.6, 0.6)	(0.9, 0.4)	(0.8, 0.4)	(0.2, 0.9)
$(e_4,q_1,0)$	(0.5, 0.4)	(0.8, 0.5)	(0.7, 0.2)	(0.5, 0.6)	(0.8, 0.3)
$(e_4,q_2,0)$	(0.8, 0.4)	(0.9, 0.3)	(0.6, 0.2)	(0.7, 0.5)	(0.5, 0.8)
$(e_4,q_3,0)$	(0.7, 0.6)	(0.8, 0.3)	(0.5, 0.8)	(0.5, 0.7)	(0.8, 0.1)

**Table 10:** Tabular representation of the FFSES $(\zeta ,B)$

$(\zeta ,B)$	$p_1$	$p_2$	$p_3$	$p_4$	$p_5$
$(e_1,q_1,1)$	(0.8, 0.2)	(0.9, 0.3)	(0.8, 0.1)	(0.2, 0.9)	(0.6, 0.5)
$(e_1,q_2,1)$	(0.4, 0.9)	(0.3, 0.7)	(0.8, 0.4)	(0.6, 0.5)	(0.4, 0.7)
$(e_1,q_3,1)$	(0.5, 0.8)	(0.7, 0.5)	(0.5, 0.4)	(0.7, 0.8)	(0.6, 0.5)
$(e_2,q_1,1)$	(0.6, 0.6)	(0.8, 0.2)	(0.7, 0.4)	(0.5, 0.7)	(0.9, 0.3)
$(e_2,q_2,1)$	(0.8, 0.3)	(0.9, 0.3)	(0.6, 0.7)	(0.6, 0.4)	(0.5, 0.8)
$(e_2,q_3,1)$	(0.9, 0.1)	(0.5, 0.4)	(0.4, 0.7)	(0.2, 0.9)	(0.6, 0.7)
$(e_3,q_1,1)$	(0.8, 0.7)	(0.9, 0.4)	(0.6, 0.6)	(0.5, 0.4)	(0.5, 0.5)
$(e_3,q_2,1)$	(0.5, 0.4)	(0.7, 0.6)	(0.8, 0.5)	(0.6, 0.5)	(0.9, 0.3)
$(e_3,q_3,1)$	(0.6, 0.2)	(0.5, 0.8)	(0.7, 0.5)	(0.8, 0.4)	(0.7, 0.6)
$(e_4,q_1,1)$	(0.8, 0.3)	(0.7, 0.3)	(0.4, 0.8)	(0.6, 0.5)	(0.8, 0.2)
$(e_4,q_2,1)$	(0.4, 0.6)	(0.8, 0.7)	(0.6, 0.5)	(0.5, 0.4)	(0.3, 0.6)
$(e_4,q_3,1)$	(0.8, 0.4)	(0.4, 0.7)	(0.6, 0.7)	(0.3, 0.6)	(0.4, 0.9)
$(e_1,q_1,0)$	(0.6, 0.3)	(0.5, 0.8)	(0.2, 0.9)	(0.6, 0.7)	(0.5, 0.5)
$(e_1,q_2,0)$	(0.5, 0.4)	(0.8, 0.3)	(0.6, 0.5)	(0.8, 0.3)	(0.2, 0.9)
$(e_1,q_3,0)$	(0.7, 0.6)	(0.9, 0.1)	(0.4, 0.7)	(0.6, 0.4)	(0.7, 0.6)
$(e_2,q_1,0)$	(0.5, 0.7)	(0.4, 0.6)	(0.8, 0.2)	(0.5, 0.8)	(0.5, 0.4)
$(e_2,q_2,0)$	(0.8, 0.1)	(0.5, 0.4)	(0.3, 0.7)	(0.5, 0.5)	(0.6, 0.3)
$(e_2,q_3,0)$	(0.9, 0.2)	(0.3, 0.6)	(0.6, 0.6)	(0.7, 0.6)	(0.8, 0.1)
$(e_3,q_1,0)$	(0.5, 0.4)	(0.7, 0.6)	(0.9, 0.4)	(0.8, 0.2)	(0.7, 0.5)
$(e_3,q_2,0)$	(0.7, 0.3)	(0.6, 0.3)	(0.5, 0.6)	(0.3, 0.7)	(0.2, 0.9)
$(e_3,q_3,0)$	(0.6, 0.7)	(0.2, 0.9)	(0.4, 0.7)	(0.9, 0.1)	(0.6, 0.5)
$(e_4,q_1,0)$	(0.5, 0.7)	(0.8, 0.2)	(0.9, 0.1)	(0.8, 0.3)	(0.4, 0.7)
$(e_4,q_2,0)$	(0.8, 0.4)	(0.3, 0.6)	(0.4, 0.7)	(0.5, 0.7)	(0.9, 0.3)
$(e_4,q_3,0)$	(0.6, 0.7)	(0.5, 0.7)	(0.8, 0.4)	(0.6, 0.4)	(0.6, 0.6)

The union of FFSESs $(\lambda ,A)$ and $(\zeta ,B)$ is given by Table 11 as follows:

**Table 11:** Tabular arrangement of the union of FFSESs $(\lambda ,A)$ and $(\zeta ,B)$

$(\lambda ,A)\Cup (\zeta ,B)$	$p_1$	$p_2$	$p_3$	$p_4$	$p_5$
$(e_1,q_1,1)$	(0.8, 0.2)	(0.9, 0.3)	(0.8, 0.1)	(0.4, 0.8)	(0.6, 0.4)
$(e_1,q_2,1)$	(0.9, 0.3)	(0.4, 0.7)	(0.8, 0.4)	(0.6, 0.5)	(0.7, 0.5)
$(e_1,q_3,1)$	(0.8, 0.7)	(0.7, 0.5)	(0.7, 0.4)	(0.7, 0.6)	(0.6, 0.5)
$(e_2,q_1,1)$	(0.6, 0.6)	(0.8, 0.2)	(0.8, 0.4)	(0.5, 0.6)	(0.9, 0.3)
$(e_2,q_2,1)$	(0.8, 0.3)	(0.9, 0.3)	(0.8, 0.4)	(0.6, 0.4)	(0.9, 0.2)
$(e_2,q_3,1)$	(0.9, 0.1)	(0.8, 0.4)	(0.6, 0.5)	(0.4, 0.6)	(0.8, 0.7)
$(e_3,q_1,1)$	(0.8, 0.4)	(0.9, 0.4)	(0.7, 0.6)	(0.8, 0.4)	(0.5, 0.5)
$(e_3,q_2,1)$	(0.7, 0.4)	(0.7, 0.6)	(0.8, 0.5)	(0.7, 0.5)	(0.9, 0.3)
$(e_3,q_3,1)$	(0.8, 0.2)	(0.5, 0.5)	(0.7, 0.5)	(0.8, 0.4)	(0.7, 0.6)
$(e_4,q_1,1)$	(0.8, 0.3)	(0.8, 0.3)	(0.4, 0.8)	(0.6, 0.5)	(0.8, 0.2)
$(e_4,q_2,1)$	(0.4, 0.6)	(0.8, 0.2)	(0.6, 0.5)	(0.7, 0.3)	(0.4, 0.6)
$(e_4,q_3,1)$	(0.8, 0.4)	(0.6, 0.4)	(0.8, 0.3)	(0.4, 0.6)	(0.9, 0.2)
$(e_1,q_1,0)$	(0.6, 0.3)	(0.8, 0.6)	(0.9, 0.2)	(0.6, 0.5)	(0.9, 0.1)
$(e_1,q_2,0)$	(0.6, 0.4)	(0.8, 0.3)	(0.6, 0.4)	(0.9, 0.3)	(0.5, 0.4)
$(e_1,q_3,0)$	(0.8, 0.6)	(0.9, 0.1)	(0.7, 0.7)	(0.6, 0.4)	(0.7, 0.6)
$(e_2,q_1,0)$	(0.6, 0.7)	(0.5, 0.6)	(0.9, 0.2)	(0.8, 0.4)	(0.7, 0.4)
$(e_2,q_2,0)$	(0.8, 0.1)	(0.7, 0.4)	(0.5, 0.3)	(0.7, 0.5)	(0.9, 0.2)
$(e_2,q_3,0)$	(0.9, 0.2)	(0.9, 0.5)	(0.8, 0.6)	(0.9, 0.6)	(0.8, 0.1)
$(e_3,q_1,0)$	(0.5, 0.4)	(0.7, 0.6)	(0.9, 0.4)	(0.9, 0.1)	(0.7, 0.5)
$(e_3,q_2,0)$	(0.9, 0.3)	(0.6, 0.3)	(0.6, 0.6)	(0.5, 0.6)	(0.9, 0.3)
$(e_3,q_3,0)$	(0.9, 0.1)	(0.6, 0.6)	(0.9, 0.4)	(0.9, 0.7)	(0.6, 0.5)
$(e_4,q_1,0)$	(0.5, 0.4)	(0.8, 0.2)	(0.9, 0.1)	(0.8, 0.3)	(0.8, 0.3)
$(e_4,q_2,0)$	(0.8, 0.4)	(0.9, 0.3)	(0.6, 0.2)	(0.7, 0.5)	(0.9, 0.3)
$(e_4,q_3,0)$	(0.7, 0.6)	(0.8, 0.3)	(0.8, 0.4)	(0.6, 0.4)	(0.8, 0.1)

### **Proposition 2**

*Let* $(\lambda ,A)$, $(\zeta ,B)$ *and* $(\xi ,D)$ *be three FFSESs over the universe P. Then*

1. $$(\lambda ,A)\Cup (\zeta ,B)=(\zeta ,B)\Cup (\lambda ,A)$$,
2. $$(\lambda ,A)\Cup {\big ((}\zeta ,B)\Cup (\xi ,D){\big )} ={\big ((}\lambda ,A)\Cup (\zeta ,B){\big )}\Cup (\xi ,D)$$.

### *Proof*

1. From Definition 14, $(\lambda ,A)\Cup (\zeta ,B)=(H_1,K_1)$ with $K_1=A\cup B$ and for all $\epsilon \in K_1$,
  $$\begin{aligned} H_1(\epsilon )= {\left\{ \begin{array}{ll} \lambda (\epsilon ),&\quad \mathrm{if}\;\epsilon \in A-B,\\ \zeta (\epsilon ),&\quad\mathrm{if}\;\epsilon \in B-A,\\ \lambda (\epsilon )\cup \zeta (\epsilon ),&\quad\mathrm{if}\;\epsilon \in A\cap B, \end{array}\right. } \end{aligned}$$
  where $\lambda (\epsilon )\cup \zeta (\epsilon )=(\max (\alpha ^1_\epsilon , \alpha ^2_\epsilon ),\min (\beta ^1_\epsilon , \beta ^2_\epsilon )),$ for all $(\alpha ^1_\epsilon ,\beta ^1_\epsilon )\in \lambda (\epsilon ),~(\alpha ^2_\epsilon ,\beta ^2_\epsilon )\in \zeta (\epsilon ).$ Similarly, by Definition 14, $(\zeta ,B)\Cup (\lambda ,A)=(H_2,K_2)$ with $K_2= B\cup A$ and for all $\epsilon \in K_2$,
  $$\begin{aligned} \begin{array}{ll} H_2(\epsilon )&= {\left\{ \begin{array}{ll} \zeta (\epsilon ),&\quad\mathrm{if}\,\epsilon \in B-A,\\ \lambda (\epsilon ),&\quad\mathrm{if}\,\epsilon \in A-B,\\ \zeta (\epsilon )\cup \lambda (\epsilon ),&\quad\mathrm{if}\,\epsilon \in B\cap A, \end{array}\right. } \\ &=H_1(\epsilon )\end{array} \end{aligned}$$
  where $\zeta (\epsilon )\cup \lambda (\epsilon )=(\max (\alpha ^1_\epsilon , \alpha ^2_\epsilon ),\min (\beta ^1_\epsilon , \beta ^2_\epsilon )),$ for all $(\alpha ^1_\epsilon ,\beta ^1_\epsilon )\in \lambda (\epsilon ),~(\alpha ^2_\epsilon ,\beta ^2_\epsilon )\in \zeta (\epsilon ).$
2. Similar to part (1). $\square$

### **Definition 15**

Let $(\lambda ,A)$ and $(\zeta ,B)$ be two FFSESs on the universe *P*. Then we define their intersection by $(I,K)=(\lambda ,A)\Cap (\zeta ,B)$, which is again a FFSES, where $K=A\cup B$ and $\forall ~\epsilon \in K$,

$$\begin{aligned} I(\epsilon )= {\left\{ \begin{array}{ll} \lambda (\epsilon ), &\quad \mathrm{if}\;\epsilon \in A-B,\\ \zeta (\epsilon ),&\quad\mathrm{if}\;\epsilon \in B-A,\\ \lambda (\epsilon )\cap \zeta (\epsilon )&\quad\mathrm{if}\;\epsilon \in A\cap B, \end{array}\right. } \end{aligned}$$

where $\lambda (\epsilon )\cup \zeta (\epsilon )=(\min (\alpha ^1_\epsilon , \alpha ^2_\epsilon ),\max (\beta ^1_\epsilon , \beta ^2_\epsilon )),$ for all $(\alpha ^1_\epsilon ,\beta ^1_\epsilon )\in \lambda (\epsilon ),~(\alpha ^2_\epsilon ,\beta ^2_\epsilon )\in \zeta (\epsilon ).$

### *Example 7*

Reconsider FFSESs $(\lambda ,A)$ and $(\zeta ,B)$ over *P* as given in Example 6. Then, their intersection $(\lambda ,A)\Cap (\zeta ,B)$ is displayed in Table 12 where

$$\begin{aligned} A&=\{(e_1,q_1,1),(e_1,q_2,1),(e_1,q_3,1),(e_2,q_1,1),(e_2,q_2,1), (e_2,q_3,1),(e_3,q_1,1),(e_3,q_2,1),\\&\qquad (e_3,q_3,1),(e_4,q_1,1),(e_4,q_2,1),(e_4,q_3,1),(e_1,q_1,0),(e_1,q_2,0), (e_1,q_3,0),(e_2,q_1,0),\\&\qquad (e_2,q_2,0),(e_2,q_3,0),(e_3,q_1,0),(e_3,q_2,0),(e_3,q_3,0),(e_4,q_1,0), (e_4,q_2,0),(e_4,q_3,0)\}\\ B&=\{(e_1,q_1,1),(e_1,q_2,1),(e_1,q_3,1),(e_2,q_1,1),(e_2,q_2,1),(e_2,q_3,1), (e_3,q_1,1),(e_3,q_2,1),\\&\qquad (e_3,q_3,1),(e_4,q_1,1),(e_4,q_2,1),(e_4,q_3,1),(e_1,q_1,0),(e_1,q_2,0), (e_1,q_3,0),(e_2,q_1,0),\\&\qquad (e_2,q_2,0),(e_2,q_3,0),(e_3,q_1,0),(e_3,q_2,0), (e_3,q_3,0),(e_4,q_1,0),(e_4,q_2,0),(e_4,q_3,0)\}\\ \end{aligned}$$

**Table 12:** Tabular representation of the intersection of FFSESs $(\lambda ,A)$ and $(\zeta ,B)$

$(\lambda ,A)\Cap (\zeta ,B)$	$p_1$	$p_2$	$p_3$	$p_4$	$p_5$
$(e_1,q_1,1)$	(0.5, 0.7)	(0.8, 0.4)	(0.7, 0.6)	(0.9, 0.8)	(0.6, 0.5)
$(e_1,q_2,1)$	(0.4, 0.9)	(0.3, 0.9)	(0.5, 0.7)	(0.4, 0.7)	(0.4, 0.7)
$(e_1,q_3,1)$	(0.5, 0.8)	(0.4, 0.7)	(0.5, 0.6)	(0.4, 0.8)	(0.4, 0.9)
$(e_2,q_1,1)$	(0.5, 0.7)	(0.7, 0.4)	(0.7, 0.4)	(0.4, 0.7)	(0.6, 0.7)
$(e_2,q_2,1)$	(0.6, 0.5)	(0.4, 0.6)	(0.6, 0.7)	(0.5, 0.4)	(0.5, 0.8)
$(e_2,q_3,1)$	(0.5, 0.7)	(0.5, 0.4)	(0.4, 0.7)	(0.2, 0.9)	(0.6, 0.7)
$(e_3,q_1,1)$	(0.8, 0.7)	(0.9, 0.4)	(0.6, 0.6)	(0.5, 0.4)	(0.5, 0.5)
$(e_3,q_2,1)$	(0.5, 0.5)	(0.6, 0.6)	(0.8, 0.5)	(0.6, 0.5)	(0.3, 0.6)
$(e_3,q_3,1)$	(0.6, 0.2)	(0.4, 0.5)	(0.7, 0.5)	(0.5, 0.4)	(0.6, 0.6)
$(e_4,q_1,1)$	(0.5, 0.6)	(0.7, 0.7)	(0.4, 0.9)	(0.4, 0.5)	(0.7, 0.3)
$(e_4,q_2,1)$	(0.2, 0.9)	(0.8, 0.7)	(0.6, 0.5)	(0.5, 0.4)	(0.3, 0.6)
$(e_4,q_3,1)$	(0.8, 0.4)	(0.4, 0.7)	(0.6, 0.7)	(0.3, 0.7)	(0.4, 0.9)
$(e_1,q_1,0)$	(0.5, 0.9)	(0.5, 0.8)	(0.2, 0.9)	(0.5, 0.7)	(0.5, 0.5)
$(e_1,q_2,0)$	(0.5, 0.5)	(0.8, 0.4)	(0.5, 0.5)	(0.8, 0.3)	(0.2, 0.9)
$(e_1,q_3,0)$	(0.7, 0.7)	(0.4, 0.4)	(0.4, 0.8)	(0.5, 0.7)	(0.4, 0.7)
$(e_2,q_1,0)$	(0.5, 0.8)	(0.4, 0.8)	(0.8, 0.3)	(0.5, 0.8)	(0.5, 0.6)
$(e_2,q_2,0)$	(0.8, 0.7)	(0.5, 0.6)	(0.3, 0.7)	(0.5, 0.6)	(0.6, 0.3)
$(e_2,q_3,0)$	(0.5, 0.8)	(0.3, 0.6)	(0.6, 0.6)	(0.7, 0.6)	(0.8, 0.7)
$(e_3,q_1,0)$	(0.5, 0.7)	(0.7, 0.8)	(0.9, 0.4)	(0.8, 0.2)	(0.7, 0.6)
$(e_3,q_2,0)$	(0.7, 0.3)	(0.5, 0.4)	(0.5, 0.8)	(0.3, 0.7)	(0.2, 0.9)
$(e_3,q_3,0)$	(0.6, 0.7)	(0.2, 0.9)	(0.4, 0.7)	(0.8, 0.7)	(0.2, 0.9)
$(e_4,q_1,0)$	(0.5, 0.7)	(0.8, 0.5)	(0.7, 0.2)	(0.5, 0.6)	(0.4, 0.7)
$(e_4,q_2,0)$	(0.8, 0.4)	(0.3, 0.6)	(0.4, 0.7)	(0.5, 0.7)	(0.5, 0.8)
$(e_4,q_3,0)$	(0.6, 0.7)	(0.5, 0.7)	(0.5, 0.8)	(0.5, 0.7)	(0.6, 0.6)

### **Proposition 3**

*Let* $(\lambda ,A)$, $(\zeta ,B)$ *and* $(\xi ,D)$ *be three FFSESs on the universe P. Then*

1. $$(\lambda ,A)\Cap (\zeta ,B)=(\zeta ,B)\Cap (\lambda ,A)$$,
2. $$(\lambda ,A)\Cap {\big ((}\zeta ,B)\Cap (\xi ,D){\big )}={\big ((}\lambda ,A)\Cap (\zeta ,B){\big )}\Cap (\xi ,D)$$.

### *Proof*

1. From Definition 14, $(\lambda ,A)\Cap (\zeta ,B)=(H_1,K_1)$ with $K_1=A\cup B$ and $\forall ~\epsilon \in K_1$,
  $$\begin{aligned} H_1(\epsilon )= {\left\{ \begin{array}{ll} \lambda (\epsilon ),&\quad\mathrm{if}\;\epsilon \in A-B,\\ \zeta (\epsilon ),&\quad\mathrm{if}\;\epsilon \in B-A,\\ \lambda (\epsilon )\cap \zeta (\epsilon ),&\quad\mathrm{if}\;\epsilon \in A\cap B, \end{array}\right. } \end{aligned}$$
  where $\lambda (\epsilon )\cap \zeta (\epsilon )=(\min (\alpha ^1_\epsilon , \alpha ^2_\epsilon ),\max (\beta ^1_\epsilon , \beta ^2_\epsilon )),$ for all $(\alpha ^1_\epsilon ,\beta ^1_\epsilon )\in \lambda (\epsilon ),~(\alpha ^2_\epsilon ,\beta ^2_\epsilon )\in \zeta (\epsilon ).$
  $$\begin{aligned} H_2(\epsilon )&= {\left\{ \begin{array}{ll} \zeta (\epsilon ),&\quad \mathrm{if}\;\epsilon \in B-A,\\ \lambda (\epsilon ), &\quad\mathrm{if}\;\epsilon \in A-B,\\ \zeta (\epsilon )\cap \lambda (\epsilon ),&\quad\mathrm{if}\;\epsilon \in B\cap A, \end{array}\right. }\\&= H_1(\epsilon ) \end{aligned}$$
  In a similar way, by Definition 14, $(\zeta ,B)\Cap (\lambda ,A)=(H_2,K_2)$ with $K_2= A\cup B$ and $\forall ~\epsilon \in K_2$, where $\zeta (\epsilon )\cap \lambda (\epsilon )=(\min (\alpha ^1_\epsilon , \alpha ^2_\epsilon ),\max (\beta ^1_\epsilon , \beta ^2_\epsilon )),$ for all $(\alpha ^1_\epsilon ,\beta ^1_\epsilon )\in \lambda (\epsilon ),~(\alpha ^2_\epsilon ,\beta ^2_\epsilon )\in \zeta (\epsilon ).$
2. Similar to part (1).

$$\square$$

### **Definition 16**

Let $(\lambda ,A)$ and $(\zeta ,B)$ be two FFSESs on the universe *P*. Then AND operation between FFSESs $(\lambda ,A)$ and $(\zeta ,B)$, represented by $(\lambda ,A)\wedge (\zeta ,B)$ and is given by

$$\begin{aligned} (\lambda ,A)\wedge (\zeta ,B)=(J,A\times B), \end{aligned}$$

where $J(\alpha ,\beta )=\lambda (\alpha )\cap \zeta (\beta ),$ for all $(\alpha ,\beta )\in A \times B$.

### *Example 8*

Suppose that $(\lambda ,A)$ and $(\zeta ,B)$ are two FFSESs on the universe $P=\{p_1,p_2,\ldots ,p_5\}$ which are respectively given by Tables 13 and 14 where

$$\begin{aligned} A&=\{(e_1,q_1,1),(e_2,q_1,1),(e_2,q_2,1),(e_2,q_3,1),(e_3,q_1,1)\},\\ B&=\{(e_3,q_3,1),(e_3,q_1,0),(e_2,q_2,1),(e_1,q_3,1)\}. \end{aligned}$$

**Table 13:** Tabular arrangement of the FFSES $(\lambda ,A)$

$(\lambda ,A)$	$p_1$	$p_2$	$p_3$	$p_4$	$p_5$
$(e_1,q_1,1)$	(0.5, 0.7)	(0.8, 0.4)	(0.7, 0.6)	(0.4, 0.8)	(0.6, 0.4)
$(e_2,q_1,1)$	(0.5, 0.7)	(0.7, 0.4)	(0.8, 0.4)	(0.4, 0.6)	(0.6, 0.7)
$(e_2,q_2,1)$	(0.6, 0.5)	(0.4, 0.6)	(0.8, 0.4)	(0.5, 0.4)	(0.9, 0.2)
$(e_2,q_3,1)$	(0.5, 0.7)	(0.8, 0.4)	(0.6, 0.5)	(0.4, 0.6)	(0.8, 0.7)
$(e_3,q_1,1)$	(0.6, 0.7)	(0.9, 0.3)	(0.6, 0.5)	(0.6, 0.6)	(0.4, 0.7)

**Table 14:** Tabular arrangement of the FFSES $( \zeta ,B)$

$( \zeta ,B)$	$p_1$	$p_2$	$p_3$	$p_4$	$p_5$
$(e_3,q_3,1)$	(0.6, 0.2)	(0.5, 0.8)	(0.7, 0.5)	(0.8, 0.4)	(0.7, 0.6)
$(e_3,q_1,0)$	(0.8, 0.7)	(0.9, 0.4)	(0.6, 0.6)	(0.5, 0.4)	(0.5, 0.5)
$(e_2,q_2,1)$	(0.8, 0.3)	(0.9, 0.3)	(0.6, 0.7)	(0.6, 0.4)	(0.5, 0.8)
$(e_1,q_3,1)$	(0.5, 0.8)	(0.7, 0.5)	(0.5, 0.4)	(0.7, 0.8)	(0.6, 0.5)

Then, the tabular form of FFSES $( \lambda ,A) \wedge (\zeta ,B )$ is displayed by Table 15.

**Table 15:** The ‘AND operation’ between FFSESs $( \lambda ,A)$ and $(\zeta ,B )$

$( \lambda ,A) \wedge (\zeta ,B )$	$p_1$	$p_2$	$p_3$	$p_4$	$p_5$
$\big ((e_1,q_1,1),(e_3,q_3,1)\big )$	(0.5, 0.7)	(0.5, 0.8)	(0.7, 0.6)	(0.4, 0.8)	(0.6, 0.6)
$\big ((e_1,q_1,1),(e_3,q_1,0)\big )$	(0.5, 0.7)	(0.8, 0.4)	(0.6, 0.6)	(0.4, 0.8)	(0.5, 0.5)
$\big ((e_1,q_1,1),(e_2,q_2,1)\big )$	(0.5, 0.7)	(0.8, 0.4)	(0.6, 0.7)	(0.4, 0.8)	(0.5, 0.8)
$\big ((e_1,q_1,1),(e_1,q_3,1)\big )$	(0.5, 0.8)	(0.7, 0.5)	(0.5, 0.6)	(0.4, 0.8)	(0.6, 0.5)
$\big ((e_2,q_1,1),(e_3,q_3,1)\big )$	(0.5, 0.7)	(0.5, 0.8)	(0.7, 0.5)	(0.4, 0.6)	(0.6, 0.7)
$\big ((e_2,q_1,1),(e_3,q_1,0)\big )$	(0.5, 0.7)	(0.7, 0.4)	(0.6, 0.6)	(0.4, 0.6)	(0.5, 0.7)
$\big ((e_2,q_1,1),(e_2,q_2,1)\big )$	(0.5, 0.7)	(0.7, 0.4)	(0.6, 0.7)	(0.4, 0.6)	(0.5, 0.8)
$\big ((e_2,q_1,1),(e_1,q_3,1)\big )$	(0.5, 0.8)	(0.7, 0.5)	(0.5, 0.4)	(0.4, 0.8)	(0.6, 0.7)
$\big ((e_2,q_2,1),(e_3,q_3,1)\big )$	(0.6, 0.5)	(0.4, 0.8)	(0.7, 0.5)	(0.5, 0.4)	(0.7, 0.6)
$\big ((e_2,q_2,1),(e_3,q_1,0)\big )$	(0.6, 0.7)	(0.4, 0.6)	(0.6, 0.6)	(0.5, 0.4)	(0.5, 0.5)
$\big ((e_2,q_2,1),(e_2,q_2,1)\big )$	(0.6, 0.5)	(0.4, 0.6)	(0.6, 0.7)	(0.5, 0.4)	(0.5, 0.8)
$\big ((e_2,q_2,1),(e_1,q_3,1)\big )$	(0.5, 0.8)	(0.4, 0.6)	(0.5, 0.4)	(0.5, 0.8)	(0.6, 0.5)
$\big ((e_2,q_3,1),(e_3,q_3,1)\big )$	(0.5, 0.7)	(0.5, 0.8)	(0.6, 0.5)	(0.4, 0.6)	(0.7, 0.7)
$\big ((e_2,q_3,1),(e_3,q_1,0)\big )$	(0.5, 0.7)	(0.8, 0.4)	(0.6, 0.6)	(0.4, 0.6)	(0.5, 0.7)
$\big ((e_2,q_3,1),(e_2,q_2,1)\big )$	(0.5, 0.7)	(0.8, 0.4)	(0.6, 0.7)	(0.4, 0.6)	(0.5, 0.8)
$\big ((e_2,q_3,1),(e_1,q_3,1)\big )$	(0.5, 0.8)	(0.7, 0.5)	(0.5, 0.5)	(0.4, 0.8)	(0.6, 0.7)
$\big ((e_3,q_1,1),(e_3,q_3,1)\big )$	(0.6, 0.7)	(0.5, 0.8)	(0.6, 0.5)	(0.6, 0.6)	(0.4, 0.7)
$\big ((e_3,q_1,1),(e_3,q_1,0)\big )$	(0.6, 0.7)	(0.9, 0.4)	(0.6, 0.6)	(0.5, 0.6)	(0.4, 0.7)
$\big ((e_3,q_1,1),(e_2,q_2,1)\big )$	(0.6, 0.7)	(0.9, 0.3)	(0.6, 0.7)	(0.6, 0.6)	(0.4, 0.8)
$\big ((e_3,q_1,1),(e_1,q_3,1)\big )$	(0.5, 0.8)	(0.7, 0.5)	(0.5, 0.5)	(0.6, 0.8)	(0.4, 0.7)

### **Definition 17**

Let $(\lambda ,A)$ and $(\zeta ,B)$ be two FFSESs over the universe *P*. Then OR operation between FFSESs $(\lambda ,A)$ and $(\zeta ,B)$, represented by $(\lambda ,A)\vee (\zeta ,B)$ and is defined by

$$\begin{aligned} (\lambda ,A)\vee (\zeta ,B)=(O,A\times B), \end{aligned}$$

where $O(\alpha ,\beta )=\lambda (\alpha )\cup \zeta (\beta ),$ for all $(\alpha ,\beta )\in A \times B$.

### *Example 9*

Reconsider Example 8. Suppose that $(\lambda ,A)$ and $(\zeta ,B)$ are two FFSESs over the universe *P* given by Tables 16 and 17, respectively, where

$$\begin{aligned} A&=\{(e_1,q_1,1),(e_2,q_1,1),(e_2,q_2,1),(e_2,q_3,1),(e_3,q_1,1)\},\\ B&=\{(e_3,q_3,1),(e_3,q_1,0),(e_2,q_2,1),(e_1,q_3,1)\}. \end{aligned}$$

**Table 16:** Tabular arrangement of the FFSES $(\lambda ,A)$

$(\lambda ,A)$	$p_1$	$p_2$	$p_3$	$p_4$	$p_5$
$(e_1,q_1,1)$	(0.5, 0.7)	(0.8, 0.4)	(0.7, 0.6)	(0.4, 0.8)	(0.6, 0.4)
$(e_2,q_1,1)$	(0.5, 0.7)	(0.7, 0.4)	(0.8, 0.4)	(0.4, 0.6)	(0.6, 0.7)
$(e_2,q_2,1)$	(0.6, 0.5)	(0.4, 0.6)	(0.8, 0.4)	(0.5, 0.4)	(0.9, 0.2)
$(e_2,q_3,1)$	(0.5, 0.7)	(0.8, 0.4)	(0.6, 0.5)	(0.4, 0.6)	(0.8, 0.7)
$(e_3,q_1,1)$	(0.6, 0.7)	(0.9, 0.3)	(0.6, 0.5)	(0.6, 0.6)	(0.4, 0.7)

**Table 17:** Tabular arrangement of the FFSES $( \zeta ,B)$

$( \zeta ,B)$	$p_1$	$p_2$	$p_3$	$p_4$	$p_5$
$(e_3,q_3,1)$	(0.6, 0.2)	(0.5, 0.8)	(0.7, 0.5)	(0.8, 0.4)	(0.7, 0.6)
$(e_3,q_1,0)$	(0.8, 0.7)	(0.9, 0.4)	(0.6, 0.6)	(0.5, 0.4)	(0.5, 0.5)
$(e_2,q_2,1)$	(0.8, 0.3)	(0.9, 0.3)	(0.6, 0.7)	(0.6, 0.4)	(0.5, 0.8)
$(e_1,q_3,1)$	(0.5, 0.8)	(0.7, 0.5)	(0.5, 0.4)	(0.7, 0.8)	(0.6, 0.5)

Then, the tabular form of FFSES $( \lambda ,A) \vee (\zeta ,B )$ is displayed by Table 18.

**Table 18:** The ‘OR operation’ between FFSESs $( \lambda ,A)$ and $(\zeta ,B )$

$( \lambda ,A) \vee (\zeta ,B )$	$p_1$	$p_2$	$p_3$	$p_4$	$p_5$
$\big ((e_1,q_1,1),(e_3,q_3,1)\big )$	(0.6, 0.2)	(0.8, 0.4)	(0.7, 0.5)	(0.8, 0.4)	(0.7, 0.4)
$\big ((e_1,q_1,1),(e_3,q_1,0)\big )$	(0.8, 0.7)	(0.9, 0.4)	(0.7, 0.6)	(0.5, 0.4)	(0.6, 0.4)
$\big ((e_1,q_1,1),(e_2,q_2,1)\big )$	(0.8, 0.3)	(0.9, 0.3)	(0.7, 0.6)	(0.6, 0.4)	(0.6, 0.4)
$\big ((e_1,q_1,1),(e_1,q_3,1)\big )$	(0.5, 0.7)	(0.8, 0.4)	(0.7, 0.4)	(0.7, 0.8)	(0.6, 0.4)
$\big ((e_2,q_1,1),(e_3,q_3,1)\big )$	(0.6, 0.2)	(0.7, 0.4)	(0.8, 0.4)	(0.8, 0.4)	(0.7, 0.6)
$\big ((e_2,q_1,1),(e_3,q_1,0)\big )$	(0.8, 0.7)	(0.9, 0.4)	(0.8, 0.4)	(0.5, 0.4)	(0.6, 0.5)
$\big ((e_2,q_1,1),(e_2,q_2,1)\big )$	(0.8, 0.3)	(0.9, 0.3)	(0.8, 0.4)	(0.6, 0.4)	(0.6, 0.7)
$\big ((e_2,q_1,1),(e_1,q_3,1)\big )$	(0.5, 0.7)	(0.7, 0.4)	(0.8, 0.4)	(0.7, 0.6)	(0.6, 0.5)
$\big ((e_2,q_2,1),(e_3,q_3,1)\big )$	(0.6, 0.2)	(0.5, 0.6)	(0.8, 0.4)	(0.8, 0.4)	(0.9, 0.2)
$\big ((e_2,q_2,1),(e_3,q_1,0)\big )$	(0.8, 0.5)	(0.9, 0.4)	(0.8, 0.4)	(0.5, 0.4)	(0.9, 0.2)
$\big ((e_2,q_2,1),(e_2,q_2,1)\big )$	(0.8, 0.3)	(0.9, 0.3)	(0.8, 0.4)	(0.6, 0.4)	(0.9, 0.2)
$\big ((e_2,q_2,1),(e_1,q_3,1)\big )$	(0.6, 0.5)	(0.7, 0.5)	(0.8, 0.4)	(0.7, 0.4)	(0.9, 0.2)
$\big ((e_2,q_3,1),(e_3,q_3,1)\big )$	(0.6, 0.2)	(0.8, 0.4)	(0.7, 0.5)	(0.8, 0.4)	(0.8, 0.6)
$\big ((e_2,q_3,1),(e_3,q_1,0)\big )$	(0.8, 0.7)	(0.9, 0.4)	(0.6, 0.5)	(0.5, 0.4)	(0.8, 0.5)
$\big ((e_2,q_3,1),(e_2,q_2,1)\big )$	(0.8, 0.3)	(0.9, 0.3)	(0.6, 0.5)	(0.6, 0.4)	(0.8, 0.7)
$\big ((e_2,q_3,1),(e_1,q_3,1)\big )$	(0.5, 0.7)	(0.8, 0.4)	(0.6, 0.4)	(0.7, 0.6)	(0.8, 0.5)
$\big ((e_3,q_1,1),(e_3,q_3,1)\big )$	(0.6, 0.2)	(0.9, 0.3)	(0.7, 0.5)	(0.8, 0.4)	(0.7, 0.6)
$\big ((e_3,q_1,1),(e_3,q_1,0)\big )$	(0.8, 0.7)	(0.9, 0.3)	(0.6, 0.5)	(0.6, 0.4)	(0.5, 0.5)
$\big ((e_3,q_1,1),(e_2,q_2,1)\big )$	(0.8, 0.3)	(0.9, 0.3)	(0.6, 0.5)	(0.6, 0.4)	(0.5, 0.7)
$\big ((e_3,q_1,1),(e_1,q_3,1)\big )$	(0.6, 0.7)	(0.9, 0.3)	(0.6, 0.4)	(0.7, 0.6)	(0.6, 0.5)

### **Proposition 4**

*Let* $(\lambda ,A)$ *and* $(\zeta ,B)$ *be two FFSESs over P. Then*

1. $$((\lambda ,A)\wedge (\zeta ,B))^c=(\lambda ,A)^c\vee (\zeta ,B)^c,$$
2. $$((\lambda ,A)\vee (\zeta ,B))^c=(\lambda ,A)^c\wedge (\zeta ,B)^c.$$

### *Proof*

Straightforward. $$\square$$

### **Proposition 5**

*Let* $(\zeta _1,A)$, $(\zeta _2,B)$ *and* $(\zeta _3,C)$ *be three FFSESs on the universe P. Then*

1. $$(\zeta _1,A)\wedge ((\zeta _2,B)\wedge (\zeta _3,C))=((\zeta _1,A)\wedge (\zeta _2,B))\wedge (\zeta _3,C)$$,
2. $$(\zeta _1,A)\vee ((\zeta _2,B)\vee (\zeta _3,C))=((\zeta _1,A)\vee (\zeta _2,B))\vee (\zeta _3,C)$$,
3. $$(\zeta _1,A)\wedge ((\zeta _2,B)\vee (\zeta _3,C))=((\zeta _1,A)\wedge (\zeta _2,B))\vee ((\zeta _1,A)\wedge (\zeta _3,C))$$,
4. $$(\zeta _1,A)\vee ((\zeta _2,B)\wedge (\zeta _3,C))=((\zeta _1,A)\vee (\zeta _2,B)\wedge ((\zeta _1,A)\vee (\zeta _3,C))$$.

### *Proof*

1. By Definition 16,
  $$\begin{aligned} (\zeta _1,A)\wedge ((\zeta _2,B,N)\wedge (\zeta _3,C,N))=(J,A\times (B\times C)), \end{aligned}$$
  where $J(\alpha ,(\beta ,\gamma ))=\zeta _1(\alpha )\cap (\zeta _2(\beta )\cap \zeta _3(\beta )),$ for all $(\alpha ,(\beta ,\gamma ))\in A\times (B \times C)$. From the Proposition 3, we get $\zeta _1(\alpha )\cap (\zeta _2(\beta )\cap \zeta _3(\beta ))=(\zeta _1(\alpha )\cap \zeta _2(\beta ))\cap \zeta _3(\beta )$. Hence, $(\zeta _1,A)\wedge ((\zeta _2,B)\wedge (\zeta _3,C))=((\zeta _1,A)\wedge (\zeta _2,B))\wedge (\zeta _3,C)$.
  Using similar arguments, parts (2-4) can be readily followed.

$$\square$$

## Application to MCGDM under FFSES

This section presents a generalized algorithm which can be used to deal with FFSES model as well as intuitionistic and Pythagorean fuzzy SESs. In MCGDM the alternatives are investigated by group of experts in the presence of multiple criterion to select the most suitable option from a set of feasible alternatives under particular conditions.

### A case study: selection of most suitable solar panel system

Photovoltaics collect energy from sun in the form of sun light and convert into electricity that can be used in building electricity, power homes, remote traffic control, boat, power at remote locations and telecommunication. In early days, solar energy used primarily for production of system to drive machinery. Module efficiency is measure how much energy the solar panel produces from the sunlight. The selection of the best solar panel system from different solar brands is always a difficult task. Every merchandiser has different needs to obtain solar panel system. Apart from the financial benefits, there are other pertinent reasons from which some are as below:

1. *Solar power uses under-utilized land *
  Using solar power, one can basically make the best use of under-utilized land and consequently produce solar energy that is a good source of electricity for everyone. Like other renewable resources, solar technology produces more reason to utilize land that has been underutilized. In several areas there are many pieces of land away from the urban areas that land can create great value if solar panel system are planted on those areas. The important fact about that land is its low price which might be option for solar energy as compared to other sources of electricity. No doubt, solar panels are used to produce solar energy in wide range. For example, recently, a 45 acre solar form has been constructed in the UK (United Kingdom), and it has ability to provide electricity for 2500 homes.
2. *Solar power is free source of energy*
  Solar energy from the sun is a very vast, inexhaustible and clean resource of power. It can be directly used for lighting homes and heating purpose. Nowadays, one of the cheapest source of power in the world is solar energy. More than 2 million solar farms have been constructed in the United States. Several manufacturers offer a solar panel. The sun provide us more power than our daily use but we are not utilizing it properly.
3. *Solar power is good for environment *
  The most common advantage of the solar power is that its a clean, green source of energy, that is, solar energy production process does not release any waste which causes global warming. Thus, it is environment friendly and safe source of solar power. The installation of solar panels systems on the roof of the house is an easy procedure. From one of the renewable resources solar energy is great for environment. Based on the analysis of the United States department of energy, the amount of sunlight received by earth in one hour is larger than the total energy utilized by the world for a whole year. The environmental advantages are the main reasons in promoting solar power projects.
  Suppose $P=\{p_1=\mathrm{Jinko~ solar} ,p_2=\mathrm{LG},p_3=\mathrm{Solar ~land},p_4=\mathrm{Mission ~solar~energy} ,p_5=\mathrm{Panasonic},p_6=\mathrm{Peimar},p_7=\mathrm{REC~ group},p_8=\mathrm{Sunpower} \}$ be the set of well known brands of solar panel system. In order to get best solar panel system consider a set of parameters $E=\{e_1=\mathrm{Unique ~performance },e_2=\mathrm{Charge ~controller}, e_3=\mathrm{Efficiency },e_4=\mathrm{Frame},e_5=\mathrm{Storage },e_6=\mathrm{Thickness }, e_7=\mathrm{Clarity },e_8=\mathrm{Cell ~temperature},e_9=\mathrm{Chemical~ reaction },e_{10}=\mathrm{Cost }, e_{11}=\mathrm{Voltage },e_{12}=\mathrm{Maximum ~power }\}$. By viewing the alternatives regarding above parameters, merchandiser wants to select the best solar panel system with the help of some experts of the domain. Let $Q=\{q_1,q_2,q_3\}$ be a set of experts (electrical engineers) to determine best quality of solar panel systems from different brands and $O=\{0,1\}$ be a set of opinion where $1= \mathrm{agree}$ and $0= \mathrm{disagree}$ and let $Y = E\times Q \times O$ and $A\subseteq Y= E\times Q \times O$. On the basis of results obtained from experts, the merchandiser constructs the following FFSES $(\lambda ,A)$ where the agree FFSES $(\lambda ,A)_1$ and dis-agree FFSES $(\lambda ,A)_0$ are given in Tables 19 and 20, respectively, where $p_{ij}$ are the entries in the tables.

**Table 19:** Tabular representation of an agree-FFSES $(\lambda ,A)_1$

$(\lambda ,A)_1$	$p_1$	$p_2$	$p_3$	$p_4$	$p_5$	$p_6$	$p_7$	$p_8$	$p_9$
$(e_1,q_1,1)$	(0.3, 0.9)	(0.7, 0.6)	(0.8, 0.5)	(0.6, 0.5)	(0.7, 0.6)	(0.8, 0.5)	(0.7, 0.5)	(0.9, 0.2)	(0.9, 0.4)
$(e_1,q_2,1)$	(0.8, 0.7)	(0.7, 0.3)	(0.9, 0.2)	(0.6, 0.2)	(0.5, 0.4)	(0.7, 0.7)	(0.6, 0.3)	(0.9, 0.2)	(0.7, 0.6)
$(e_1,q_3,1)$	(0.8, 0.5)	(0.9, 0.4)	(0.6, 0.5)	(0.8, 0.5)	(0.7, 0.5)	(0.7, 0.6)	(0.5, 0.4)	(0.7, 0.3)	(0.9, 0.4)
$(e_2,q_1,1)$	(0.7, 0.4)	(0.6, 0.5)	(0.5, 0.4)	(0.8, 0.7)	(0.9, 0.2)	(0.5, 0.4)	(0.8, 0.7)	(0.6, 0.3)	(0.6, 0.5)
$(e_2,q_2,1)$	(0.7, 0.4)	(0.8, 0.2)	(0.6, 0.6)	(0.4, 0.9)	(0.8, 0.5)	(0.5, 0.4)	(0.6, 0.3)	(0.5, 0.4)	(0.7, 0.6)
$(e_2,q_3,1)$	(0.6, 0.4)	(0.8, 0.4)	(0.9, 0.3)	(0.7, 0.6)	(0.4.0.8)	(0.6, 0.4)	(0.7, 0.5)	(0.6, 0.5)	(0.5, 0.7)
$(e_3,q_1,1)$	(0.8, 0.7)	(0.6, 0.8)	(0.9, 0.4)	(0.8, 0.6)	(0.2, 0.9)	(0.6, 0.8)	(0.9, 0.3)	(0.5, 0.4)	(0.8, 0.7)
$(e_3,q_2,1)$	(0.8, 0.4)	(0.5, 0.7)	(0.8, 0.1)	(0.9, 0.4)	(0.9, 0.1)	(0.7, 0.6)	(0.9, 0.1)	(0.6, 0.5)	(0.8, 0.4)
$(e_3,q_3,1)$	(0.8, 0.2)	(0.9, 0.3)	(0.8, 0.1)	(0.2, 0.9)	(0.6, 0.5)	(0.4, 0.7)	(0.6, 0.5)	(0.8, 0.4)	(0.3, 0.7)
$(e_4,q_1,1)$	(0.5, 0.8)	(0.7, 0.5)	(0.5, 0.4)	(0.7, 0.8)	(0.6, 0.5)	(0.7, 0.8)	(0.5, 0.4)	(0.7, 0.5)	(0.6, 0.5)
$(e_4,q_2,1)$	(0.6, 0.7)	(0.8, 0.4)	(0.5, 0.7)	(0.6, 0.4)	(0.9, 0.3)	(0.4, 0.7)	(0.5, 0.7)	(0.3, 0.6)	(0.5, 0.7)
$(e_4,q_3,1)$	(0.6, 0.7)	(0.2, 0.9)	(0.4, 0.7)	(0.9, 0.1)	(0.5, 0.4)	(0.6, 0.3)	(0.7, 0.5)	(0.4, 0.6)	(0.9, 0.2)
$(e_5,q_1,1)$	(0.9, 0.3)	(0.3, 0.7)	(0.7, 0.5)	(0.8, 0.2)	(0.9, 0.3)	(0.5, 0.4)	(0.9, 0.4)	(0.7, 0.6)	(0.5, 0.8)
$(e_5,q_2,1)$	(0.5, 0.4)	(0.7, 0.6)	(0.5, 0.7)	(0.8, 0.1)	(0.9, 0.2)	(0.5, 0.4)	(0.7, 0.3)	(0.6, 0.7)	(0.5, 0.7)
$(e_5,q_3,1)$	(0.5, 0.4)	(0.8, 0.3)	(0.6, 0.5)	(0.8, 0.3)	(0.2, 0.9)	(0.6, 0.7)	(0.3, 0.6)	(0.5, 0.4)	(0.2, 0.9)
$(e_6,q_1,1)$	(0.5, 0.8)	(0.8, 0.4)	(0.7, 0.5)	(0.9, 0.4)	(0.3, 0.6)	(0.4, 0.7)	(0.8, 0.2)	(0.9, 0.3)	(0.6, 0.7)
$(e_6,q_2,1)$	(0.8, 0.5)	(0.7, 0.4)	(0.9, 0.1)	(0.6, 0.4)	(0.5, 0.4)	(0.2, 0.9)	(0.8, 0.4)	(0.9, 0.2)	(0.5, 0.4)
$(e_6,q_3,1)$	(0.8, 0.7)	(0.5, 0.7)	(0.9, 0.3)	(0.5, 0.4)	(0.8, 0.4)	(0.7, 0.6)	(0.5, 0.8)	(0.2, 0.9)	(0.8, 0.3)
$(e_7,q_1,1)$	(0.8, 0.4)	(0.6, 0.3)	(0.8, 0.3)	(0.9, 0.3)	(0.5, 0.8)	(0.5, 0.3)	(0.4, 0.7)	(0.5, 0.4)	(0.7, 0.8)
$(e_7,q_2,1)$	(0.7, 0.5)	(0.8, 0.2)	(0.9, 0.3)	(0.8, 0.4)	(0.9, 0.4)	(0.7, 0.6)	(0.5, 0.6)	(0.8, 0.3)	(0.7, 0.2)
$(e_7,q_3,1)$	(0.5, 0.6)	(0.7, 0.4)	(0.9, 0.5)	(0.7, 0.6)	(0.6, 0.3)	(0.8, 0.2)	(0.9, 0.3)	(0.8, 0.3)	(0.7, 0.6)
$(e_8,q_1,1)$	(0.8, 0.2)	(0.9, 0.3)	(0.8, 0.7)	(0.7, 0.3)	(0.9, 0.1)	(0.8, 0.4)	(0.7, 0.3)	(0.8, 0.3)	(0.6, 0.4)
$(e_8,q_2,1)$	(0.9, 0.3)	(0.4, 0.7)	(0.7, 0.5)	(0.8, 0.2)	(0.9, 0.3)	(0.8, 0.4)	(0.7, 0.6)	(0.8, 0.3)	(0.7, 0.4)
$(e_8,q_3,1)$	(0.8, 0.4)	(0.7, 0.3)	(0.9, 0.1)	(0.6, 0.4)	(0.3, 0.9)	(0.4, 0.8)	(0.7, 0.2)	(0.6, 0.5)	(0.7, 0.6)
$(e_9,q_1,1)$	(0.6, 0.4)	(0.8, 0.4)	(0.9, 0.2)	(0.8, 0.1)	(0.7, 0.5)	(0.9, 0.3)	(0.8, 0.1)	(0.6, 0.5)	(0.7, 0.4)
$(e_9,q_2,1)$	(0.8, 0.3)	(0.5, 0.7)	(0.9, 0.2)	(0.5, 0.4)	(0.9, 0.3)	(0.8, 0.4)	(0.7, 0.5)	(0.9, 0.4)	(0.9, 0.5)
$(e_9,q_3,1)$	(0.8, 0.4)	(0.6, 0.5)	(0.7, 0.6)	(0.8, 0.5)	(0.4, 0.7)	(0.9, 0.2)	(0.5, 0.3)	(0.8, 0.6)	(0.3, 0.8)
$(e_{10},q_1,1)$	(0.6, 0.7)	(0.8, 0.1)	(0.9, 0.2)	(0.5, 0.4)	(0.8, 0.4)	(0.7, 0.6)	(0.6, 0.2)	(0.7, 0.5)	(0.7, 0.4)
$(e_{10},q_2,1)$	(0.7, 0.5)	(0.8, 0.3)	(0.5, 0.4)	(0.7, 0.6)	(0.8, 0.4)	(0.9, 0.1)	(0.3, 0.7)	(0.2, 0.9)	(0.8, 0.4)
$(e_{10},q_3,1)$	(0.9, 0.3)	(0.5, 0.8)	(0.6, 0.7)	(0.7, 0.6)	(0.2, 0.9)	(0.7, 0.5)	(0.4, 0.6)	(0.9, 0.1)	(0.7, 0.6)
$(e_{11},q_1,1)$	(0.9, 0.3)	(0.5, 0.4)	(0.7, 0.6)	(0.5, 0.8)	(0.7, 0.3)	(0.6, 0.4)	(0.5, 0.7)	(0.3, 0.6)	(0.4, 0.7)
$(e_{11},q_2,1)$	(0.4, 0.9)	(0.7, 0.6)	(0.8, 0.4)	(0.5, 0.7)	(0.8, 0.4)	(0.7, 0.5)	(0.7, 0.8)	(0.8, 0.2)	(0.5, 0.8)
$(e_{11},q_3,1)$	(0.4, 0.7)	(0.5, 0.8)	(0.8, 0.3)	(0.9, 0.1)	(0.4, 0.6)	(0.5, 0.4)	(0.7, 0.3)	(0.2, 0.9)	(0.8, 0.2)
$(e_{12},q_1,1)$	(0.7, 0.4)	(0.6, 0.7)	(0.4, 0.7)	(0.8, 0.5)	(0.4, 0.7)	(0.6, 0.3)	(0.9, 0.1)	(0.6, 0.7)	(0.8, 0.1)
$(e_{12},q_2,1)$	(0.9, 0.3)	(0.8, 0.4)	(0.6, 0.5)	(0.8, 0.3)	(0.5, 0.8)	(0.6, 0.4)	(0.9, 0.4)	(0.6, 0.5)	(0.2, 0.9)
$(e_{12},q_3,1)$	(0.7, 0.5)	(0.8, 0.4)	(0.6, 0.5)	(0.3, 0.9)	(0.8, 0.1)	(0.6, 0.3)	(0.4, 0.9)	(0.6, 0.7)	(0.8, 0.4)

**Table 20:** Tabular representation of a disagree-FFSES $(\lambda ,A)_0$

$(\lambda ,A)_0$	$p_1$	$p_2$	$p_3$	$p_4$	$p_5$	$p_6$	$p_7$	$p_8$	$p_9$
$(e_1,q_1,0)$	(0.8, 0.3)	(0.5, 0.4)	(0.7, 0.7)	(0.6, 0.3)	(0.9, 0.2)	(0.7, 0.6)	(0.6, 0.5)	(0.3, 0.9)	(0.8, 0.1)
$(e_1,q_2,0)$	(0.6, 0.2)	(0.7, 0.5)	(0.7, 0.4)	(0.8, 0.3)	(0.5, 0.7)	(0.9, 0.2)	(0.5, 0.4)	(0.9, 0.3)	(0.8, 0.4)
$(e_1,q_3,0)$	(0.8, 0.4)	(0.7, 0.3)	(0.9, 0.1)	(0.6, 0.4)	(0.3, 0.9)	(0.4, 0.8)	(0.7, 0.2)	(0.6, 0.5)	(0.7, 0.6)
$(e_2,q_1,0)$	(0.7, 0.4)	(0.6, 0.2)	(0.7, 0.3)	(0.9, 0.1)	(0.7, 0.5)	(0.8, 0.3)	(0.9, 0.1)	(0.4, 0.6)	(0.9, 0.3)
$(e_2,q_2,0)$	(0.7, 0.6)	(0.6, 0.5)	(0.4, 0.7)	(0.5, 0.8)	(0.8, 0.3)	(0.9, 0.1)	(0.4, 0.6)	(0.5, 0.4)	(0.7, 0.3)
$(e_2,q_3,0)$	(0.2, 0.9)	(0.8, 0.2)	(0.8, 0.4)	(0.6, 0.3)	(0.7, 0.5)	(0.7, 0.6)	(0.6, 0.5)	(0.8, 0.4)	(0.6, 0.5)
$(e_3,q_1,0)$	(0.3, 0.9)	(0.8, 0.1)	(0.6, 0.3)	(0.4, 0.9)	(0.6, 0.7)	(0.8, 0.4)	(0.9, 0.1)	(0.3, 0.7)	(0.2, 0.9)
$(e_3,q_2,0)$	(0.7, 0.5)	(0.8, 0.3)	(0.7, 0.6)	(0.6, 0.5)	(0.4, 0.7)	(0.5, 0.4)	(0.7, 0.6)	(0.6, 0.4)	(0.7, 0.3)
$(e_3,q_3,0)$	(0.8, 0.4)	(0.6, 0.7)	(0.8, 0.4)	(0.7, 0.5)	(0.9, 0.1)	(0.8, 0.4)	(0.6, 0.5)	(0.8, 0.1)	(0.6, 0.4)
$(e_4,q_1,0)$	(0.7, 0.4)	(0.5, 0.7)	(0.8, 0.1)	(0.9, 0.4)	(0.9, 0.1)	(0.7, 0.6)	(0.9, 0.1)	(0.6, 0.5)	(0.9, 0.4)
$(e_4,q_2,0)$	(0.9, 0.2)	(0.7, 0.3)	(0.6, 0.4)	(0.5, 0.7)	(0.3, 0.6)	(0.4, 0.7)	(0.5, 0.2)	(0.7, 0.6)	(0.2, 0.9)
$(e_4,q_3,0)$	(0.4, 0.7)	(0.9, 0.1)	(0.2, 0.9)	(0.5, 0.4)	(0.6, 0.3)	(0.9, 0.2)	(0.6, 0.7)	(0.7, 0.5)	(0.4, 0.6)
$(e_5,q_1,0)$	(0.3, 0.7)	(0.7, 0.5)	(0.8, 0.2)	(0.9, 0.3)	(0.5, 0.4)	(0.9, 0.4)	(0.7, 0.6)	(0.5, 0.8)	(0.9, 0.3)
$(e_5,q_2,0)$	(0.5, 0.4)	(0.7, 0.6)	(0.5, 0.7)	(0.8, 0.1)	(0.9, 0.2)	(0.5, 0.4)	(0.7, 0.3)	(0.6, 0.7)	(0.5, 0.7)
$(e_5,q_3,0)$	(0.6, 0.7)	(0.3, 0.6)	(0.5, 0.4)	(0.2, 0.9)	(0.5, 0.4)	(0.8, 0.3)	(0.6, 0.5)	(0.8, 0.3)	(0.2, 0.9)
$(e_6,q_1,0)$	(0.5, 0.8)	(0.8, 0.4)	(0.7, 0.5)	(0.9, 0.4)	(0.3, 0.6)	(0.4, 0.7)	(0.8, 0.2)	(0.9, 0.3)	(0.6, 0.7)
$(e_6,q_2,0)$	(0.9, 0.2)	(0.8, 0.5)	(0.7, 0.4)	(0.9, 0.1)	(0.6, 0.4)	(0.5, 0.4)	(0.8, 0.4)	(0.5, 0.4)	(0.9, 0.3)
$(e_6,q_3,0)$	(0.5, 0.8)	(0.8, 0.7)	(0.5, 0.7)	(0.9, 0.3)	(0.5, 0.4)	(0.8, 0.4)	(0.7, 0.6)	(0.2, 0.9)	(0.8, 0.3)
$(e_7,q_1,0)$	(0.3, 0.6)	(0.5, 0.3)	(0.8, 0.4)	(0.6, 0.3)	(0.8, 0.3)	(0.5, 0.4)	(0.7, 0.8)	(0.9, 0.3)	(0.4, 0.7)
$(e_7,q_2,0)$	(0.8, 0.4)	(0.9, 0.4)	(0.7, 0.2)	(0.7, 0.5)	(0.8, 0.2)	(0.9, 0.3)	(0.7, 0.6)	(0.5, 0.6)	(0.8, 0.3)
$(e_7,q_3,0)$	(0.6, 0.3)	(0.8, 0.2)	(0.5, 0.6)	(0.7, 0.4)	(0.9, 0.5)	(0.7, 0.6)	(0.9, 0.3)	(0.8, 0.3)	(0.7, 0.6)
$(e_8,q_1,0)$	(0.9, 0.1)	(0.8, 0.4)	(0.7, 0.3)	(0.8, 0.3)	(0.6, 0.4)	(0.8, 0.2)	(0.9, 0.3)	(0.8, 0.7)	(0.7, 0.3)
$(e_8,q_2,0)$	(0.9, 0.3)	(0.7, 0.4)	(0.8, 0.4)	(0.9, 0.3)	(0.4, 0.7)	(0.7, 0.5)	(0.8, 0.2)	(0.7, 0.6)	(0.8, 0.3)
$(e_8,q_3,0)$	(0.7, 0.2)	(0.6, 0.5)	(0.7, 0.6)	(0.3, 0.9)	(0.4, 0.8)	(0.8, 0.4)	(0.7, 0.3)	(0.9, 0.1)	(0.6, 0.4)
$(e_9,q_1,0)$	(0.9, 0.2)	(0.8, 0.1)	(0.7, 0.5)	(0.9, 0.3)	(0.8, 0.1)	(0.6, 0.5)	(0.7, 0.4)	(0.6, 0.4)	(0.8, 0.4)
$(e_9,q_2,0)$	(0.8, 0.4)	(0.7, 0.5)	(0.9, 0.4)	(0.9, 0.5)	(0.8, 0.3)	(0.5, 0.7)	(0.9, 0.2)	(0.5, 0.4)	(0.9, 0.3)
$(e_9,q_3,0)$	(0.6, 0.5)	(0.9, 0.2)	(0.5, 0.3)	(0.8, 0.6)	(0.3, 0.8)	(0.4, 0.7)	(0.7, 0.5)	(0.5, 0.7)	(0.9, 0.2)
$(e_{10},q_1,0)$	(0.8, 0.3)	(0.5, 0.8)	(0.6, 0.4)	(0.9, 0.4)	(0.6, 0.5)	(0.2, 0.9)	(0.9, 0.3)	(0.4, 0.7)	(0.7, 0.5)
$(e_{10},q_2,0)$	(0.7, 0.5)	(0.8, 0.3)	(0.5, 0.4)	(0.7, 0.6)	(0.8, 0.4)	(0.9, 0.1)	(0.3, 0.7)	(0.2, 0.9)	(0.8, 0.4)
$(e_{10},q_3,0)$	(0.2, 0.9)	(0.5, 0.7)	(0.5, 0.5)	(0.5, 0.5)	(0.8, 0.4)	(0.5, 0.5)	(0.8, 0.3)	(0.2, 0.9)	(0.8, 0.4)
$(e_{11},q_1,0)$	(0.5, 0.5)	(0.6, 0.6)	(0.8, 0.5)	(0.6, 0.5)	(0.3, 0.6)	(0.6, 0.2)	(0.4, 0.5)	(0.7, 0.5)	(0.5, 0.4)
$(e_{11},q_2,0)$	(0.6, 0.6)	(0.5, 0.6)	(0.7, 0.7)	(0.4, 0.9)	(0.4, 0.5)	(0.7, 0.3)	(0.2, 0.9)	(0.8, 0.7)	(0.6, 0.5)
$(e_{11},q_3,0)$	(0.5, 0.4)	(0.3, 0.6)	(0.8, 0.4)	(0.4, 0.7)	(0.6, 0.7)	(0.3, 0.7)	(0.4, 0.9)	(0.5, 0.9)	(0.5, 0.8)
$(e_{12},q_1,0)$	(0.8, 0.4)	(0.9, 0.1)	(0.3, 0.7)	(0.7, 0.6)	(0.6, 0.5)	(0.4, 0.7)	(0.5, 0.8)	(0.6, 0.4)	(0.7, 0.3)
$(e_{12},q_2,0)$	(0.3, 0.9)	(0.8, 0.1)	(0.6, 0.3)	(0.4, 0.9)	(0.7, 0.6)	(0.5, 0.7)	(0.8, 0.4)	(0.7, 0.4)	(0.6, 0.7)
$(e_{12},q_3,0)$	(0.6, 0.2)	(0.5, 0.4)	(0.7, 0.7)	(0.6, 0.3)	(0.9, 0.2)	(0.5, 0.6)	(0.8, 0.3)	(0.7, 0.2)	(0.9, 0.1)

Now the agree- and disagree-score tables are given by Tables 21 and 22, respectively.

**Table 21:** Agree-score table of FFSES $(\lambda ,A)_1$

$(\lambda ,A)_1$	$p_1$	$p_2$	$p_3$	$p_4$	$p_5$	$p_6$	$p_7$	$p_8$	$p_9$
$(e_1,q_1,1)$	− 0.702	0.127	0.387	0.091	0.127	0.387	0.218	0.721	0.665
$(e_1,q_2,1)$	0.169	0.316	0.721	0.208	0.061	0	0.189	0.721	0.127
$(e_1,q_3,1)$	0.387	0.665	0.091	0.387	0.218	0.127	0.061	0.316	0.665
$(e_2,q_1,1)$	0.279	0.091	0.061	0.169	0.721	0.061	0.169	0.189	0.091
$(e_2,q_2,1)$	0.279	0.504	0	− 0.665	0.387	0.061	0.189	0.061	0.127
$(e_2,q_3,1)$	0.152	0.448	0.702	0.127	− 0.448	0.152	0.218	0.091	− 0.218
$(e_3,q_1,1)$	0.169	− 0.296	0.665	0.296	− 0.721	− 0.296	0.702	0.061	0.169
$(e_3,q_2,1)$	0.448	− 0.218	0.511	0.665	0.728	0.127	0.728	0.091	0.448
$(e_3,q_3,1)$	0.504	0.702	0.511	− 0.721	0.091	− 0.279	0.091	0.448	− 0.316
$(e_4,q_1,1)$	− 0.387	0.218	0.061	− 0.169	0.091	− 0.169	0.061	0.218	0.091
$(e_4,q_2,1)$	− 0.127	0.448	− 0.218	0.152	0.702	− 0.279	− 0.218	− 0.189	− 0.218
$(e_4,q_3,1)$	− 0.127	− 0.721	− 0.279	0.728	0.061	0.189	0.218	− 0.152	0.721
$(e_5,q_1,1)$	0.702	− 0.316	0.218	0.504	0.702	0.061	0.665	0.127	− 0.387
$(e_5,q_2,1)$	0.061	0.127	− 0.218	0.511	0.721	0.061	0.316	− 0.127	− 0.218
$(e_5,q_3,1)$	0.061	0.485	0.091	0.485	− 0.721	− 0.127	− 0.189	0.061	− 0.721
$(e_6,q_1,1)$	− 0.387	0.448	0.218	0.665	− 0.189	− 0.279	0.504	0.702	− 0.127
$(e_6,q_2,1)$	0.387	0.279	0.728	0.152	0.061	− 0.721	0.448	0.721	0.061
$(e_6,q_3,1)$	0.169	− 0.218	0.702	0.061	0.448	0.127	− 0.387	− 0.721	0.485
$(e_7,q_1,1)$	0.448	0.189	0.485	0.702	− 0.387	0.098	− 0.279	0.061	− 0.169
$(e_7,q_2,1)$	0.218	0.504	0.702	0.448	0.665	0.127	− 0.091	0.485	0.335
$(e_7,q_3,1)$	− 0.091	0.279	0.604	0.127	0.189	0.504	0.702	0.485	0.127
$(e_8,q_1,1)$	0.504	0.702	0.169	0.316	0.728	0.448	0.316	0.485	0.152
$(e_8,q_2,1)$	0.702	− 0.279	0.218	0.504	0.702	0.448	0.127	0.485	0.279
$(e_8,q_3,1)$	0.448	0.316	0.728	0.152	− 0.702	− 0.448	0.335	0.091	0.127
$(e_9,q_1,1)$	0.152	0.448	0.721	0.511	0.218	0.702	0.511	0.091	0.279
$(e_9,q_2,1)$	0.485	− 0.218	0.721	0.061	0.702	0.448	0.218	0.665	0.604
$(e_9,q_3,1)$	0.448	0.091	0.127	0.387	− 0.279	0.721	0.098	0.296	− 0.485
$(e_{10},q_1,1)$	− 0.127	0.511	0.721	0.061	0.448	0.127	0.208	0.218	0.279
$(e_{10},q_2,1)$	0.218	0.485	0.061	0.127	0.448	0.728	− 0.316	− 0.721	0.448
$(e_{10},q_3,1)$	0.702	− 0.387	− 0.127	0.127	− 0.721	0.218	− 0.152	0.728	0.127
$(e_{11},q_1,1)$	0.702	0.061	0.127	− 0.387	0.316	0.152	− 0.218	− 0.189	− 0.279
$(e_{11},q_2,1)$	− 0.665	0.127	0.448	− 0.218	0.448	0.218	− 0.169	0.504	− 0.387
$(e_{11},q_3,1)$	− 0.279	− 0.387	0.485	0.728	− 0.152	0.061	0.316	− 0.721	0.504
$(e_{12},q_1,1)$	0.279	− 0.127	− 0.279	0.387	− 0.279	0.189	0.728	− 0.127	0.511
$(e_{12},q_2,1)$	0.702	0.448	0.091	0.485	− 0.387	0.152	0.665	0.091	− 0.721
$(e_{12},q_3,1)$	0.218	0.448	0.091	− 0.702	0.511	0.189	− 0.665	− 0.127	0.448
$a_j=\sum _i p_{ij}$	$a_1=7.101$	$a_2=6.3$	$a_3=11.045$	$a_4=7.462$	$a_5=5.508$	$a_6=3.837$	$a_7=6.317$	$a_8=6.139$	$a_9=3.624$

**Table 22:** Disagree-score table of FFSES $(\lambda ,A)_0$

$(\lambda ,A)_0$	$p_1$	$p_2$	$p_3$	$p_4$	$p_5$	$p_6$	$p_7$	$p_8$	$p_9$
$(e_1,q_1,0)$	0.485	0.061	0	0.189	0.721	0.127	0.091	− 0.702	0.511
$(e_1,q_2,0)$	0.208	0.218	0.279	0.485	− 0.218	0.721	0.061	0.702	0.448
$(e_1,q_3,0)$	0.448	0.316	0.728	0.152	− 0.702	− 0.448	0.335	0.091	0.127
$(e_2,q_1,0)$	0.279	0.208	0.316	0.728	0.218	0.485	0.728	− 0.152	0.702
$(e_2,q_2,0)$	0.127	0.091	− 0.279	− 0.387	0.485	0.728	− 0.152	0.061	0.316
$(e_2,q_3,0)$	− 0.721	0.504	0.448	0.189	0.218	0.127	0.091	0.448	0.091
$(e_3,q_1,0)$	− 0.702	0.511	0.189	− 0.665	− 0.127	0.448	0.728	− 0.316	− 0.721
$(e_3,q_2,0)$	0.218	0.485	0.127	0.091	− 0.279	0.061	0.127	0.152	0.316
$(e_3,q_3,0)$	0.448	− 0.127	0.448	0.218	0.728	0.448	0.091	0.511	0.152
$(e_4,q_1,0)$	0.279	− 0.218	0.511	0.665	0.728	0.127	0.728	0.091	0.665
$(e_4,q_2,0)$	0.721	0.316	0.152	− 0.218	− 0.189	− 0.279	0.117	0.127	− 0.721
$(e_4,q_3,0)$	− 0.279	0.728	− 0.721	0.061	0.189	0.721	− 0.127	0.218	− 0.152
$(e_5,q_1,0)$	− 0.316	0.218	0.504	0.702	0.061	0.665	0.127	− 0.387	0.702
$(e_5,q_2,0)$	0.061	0.127	− 0.218	0.511	0.721	0.061	0.316	− 0.127	− 0.218
$(e_5,q_3,0)$	− 0.127	− 0.189	0.061	− 0.721	0.061	0.485	0.091	0.485	− 0.721
$(e_6,q_1,0)$	− 0.387	0.448	0.218	0.665	− 0.189	− 0.279	0.504	0.702	− 0.127
$(e_6,q_2,0)$	0.721	0.387	0.279	0.728	0.152	0.061	0.448	0.061	0.702
$(e_6,q_3,0)$	− 0.387	0.169	− 0.218	0.702	0.061	0.448	0.127	− 0.721	0.485
$(e_7,q_1,0)$	− 0.189	0.098	0.448	0.189	0.485	0.061	− 0.169	0.702	− 0.279
$(e_7,q_2,0)$	0.448	0.665	0.335	0.218	0.504	0.702	0.127	− 0.091	0.485
$(e_7,q_3,0)$	0.189	0.504	− 0.091	0.279	0.604	0.127	0.702	0.485	0.127
$(e_8,q_1,0)$	0.728	0.448	0.316	0.485	0.152	0.504	0.702	0.169	0.316
$(e_8,q_2,0)$	0.702	0.279	0.448	0.702	− 0.279	0.218	0.504	0.127	0.485
$(e_8,q_3,0)$	0.335	0.091	0.127	− 0.702	− 0.448	0.448	0.316	0.728	0.152
$(e_9,q_1,0)$	0.721	0.511	0.218	0.702	0.511	0.091	0.279	0.152	0.448
$(e_9,q_2,0)$	0.448	0.218	0.665	0.604	0.485	− 0.218	0.721	0.061	0.702
$(e_9,q_3,0)$	0.091	0.721	0.098	0.296	− 0.485	− 0.279	0.218	− 0.218	0.721
$(e_{10},q_1,0)$	0.485	− 0.387	0.152	0.665	0.091	− 0.721	0.702	− 0.279	0.218
$(e_{10},q_2,0)$	0.218	0.485	0.061	0.127	0.448	0.728	− 0.316	− 0.721	0.448
$(e_{10},q_3,0)$	− 0.721	− 0.218	0	0	0.448	0	0.485	− 0.721	0.448
$(e_{11},q_1,0)$	0	0	0.387	0.091	− 0.189	0.208	− 0.061	0.218	0.061
$(e_{11},q_2,0)$	0	− 0.091	0	− 0.665	− 0.061	0.316	− 0.721	0.169	0.091
$(e_{11},q_3,0)$	0.061	− 0.189	0.448	− 0.279	− 0.127	− 0.316	− 0.665	− 0.604	− 0.387
$(e_{12},q_1,0)$	0.448	0.728	− 0.316	0.127	0.091	− 0.279	− 0.387	0.152	0.316
$(e_{12},q_2,0)$	− 0.702	0.511	0.189	− 0.665	0.127	− 0.218	0.448	0.279	− 0.127
$(e_{12},q_3,0)$	0.208	0.061	0	0.189	0.721	− 0.091	0.485	0.335	0.728
$b_j=\sum _i p_{ij}$	$b_1= 4.546$	$b_2=8.688$	$b_3=6.030$	$b_4=6.458$	$b_5=4.935$	$b_6=5.988$	$b_7=7.801$	$b_8= 2.187$	$b_9=7.510$

In the following, we develop an algorithm (see Algorithm 1) which is used in the selection of most suitable solar panel system. We establish it in the language of our running example but it can be naturally adapted to the exact formulations of other MCGDM problems: 
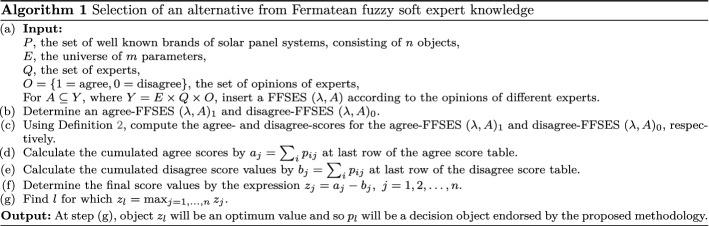


Now by applying Algorithm 1, we find the best alternative for vendor of solar panel system. Table 23 can be readily obtained from Tables 21 and 22.

**Table 23:** Final score table

$a_j=\sum _i p_{ij}$	$b_j=\sum _i p_{ij}$	$z_j=a_j-b_j$
$a_1=7.101$	$b_1=4.546$	$z_1=2.555$
$a_2=6.300$	$b_2=8.688$	$z_2=-2.388$
$a_3=11.045$	$b_3=6.030$	$z_3=5.015$
$a_4=7.462$	$b_4=6.458$	$z_4=1.004$
$a_5=5.508$	$b_5=4.935$	$z_5=0.573$
$a_6=3.837$	$b_6=5.988$	$z_6=-2.151$
$a_7=6.317$	$b_7=7.801$	$z_7=-1.484$
$a_8=6.139$	$b_8=2.187$	$z_8=3.952$
$a_9=3.624$	$b_9=7.510$	$z_9=-3.886$

From the above Table 23, one can easily see that $\max z_j=z_3$, so the alternative $p_3$ will be selected as best brand to purchase solar panel system for the vendor.

There may be situations for which different weights are assigned by the experts to different parameters. This improvement should make the selection process more effective. Now we revise Algorithm 1 accordingly. This enhancement is based on the needs of the experts in the selection procedure of the most suitable solar panel system.
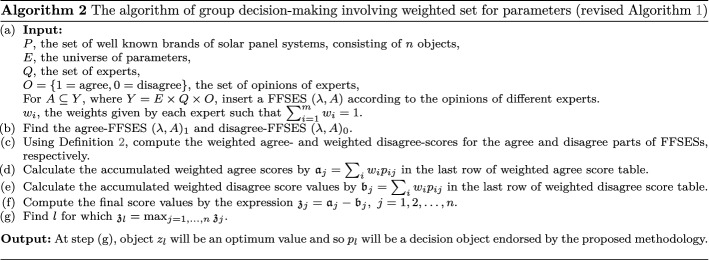


Let us now apply Algorithm 2 on the above Application. To this purpose we consider the weights proposed by the experts which are displayed in Table 24.

**Table 24:** Weights provided by experts

Experts	$e_1$	$e_2$	$e_3$	$e_4$	$e_5$	$e_6$	$e_7$	$e_8$	$e_9$	$e_{10}$	$e_{11}$	$e_{12}$
$q_1$	0.05	0.02	0.23	0.11	0.09	0.12	0.03	0.04	0.06	0.07	0.14	0.04
$q_2$	0.15	0.07	0.13	0.07	0.04	0.10	0.02	0.01	0.08	0.09	0.13	0.11
$q_3$	0.10	0.05	0.17	0.08	0.07	0.09	0.01	0.03	0.05	0.04	0.19	0.12

Then the weighted agree score table is provided by Table 25.

**Table 25:** Weighted agree-score table of FFSES $(\lambda ,A)_1$

$(\lambda ,A)_1$	$p_1$	$p_2$	$p_3$	$p_4$	$p_5$	$p_6$	$p_7$	$p_8$	$p_9$
$(e_1,q_1,1)$	− 0.0351	0.0064	0.0194	0.0046	0.0064	0.0194	0.0109	0.0360	0.0333
$(e_1,q_2,1)$	0.0254	0.0474	0.1082	0.0312	0.0092	0	0.0284	0.1082	0.0191
$(e_1,q_3,1)$	0.0387	0.0665	0.0091	0.0387	0.0218	0.0127	0.0061	0.0316	0.0665
$(e_2,q_1,1)$	0.0056	0.0018	0.0012	0.0034	0.0144	0.0012	0.0034	0.0038	0.0018
$(e_2,q_2,1)$	0.0195	0.0353	0	− 0.0466	0.0271	0.0043	0.0132	0.0043	0.0089
$(e_2,q_3,1)$	0.0076	0.0224	0.0351	0.0064	− 0.0224	0.0076	0.0109	0.0046	− 0.0109
$(e_3,q_1,1)$	0.0389	− 0.0681	0.1530	0.0681	− 0.1658	− 0.0681	0.1615	0.0140	0.0389
$(e_3,q_2,1)$	0.0582	− 0.0283	0.0664	0.0865	0.0946	0.0165	0.0946	0.0118	0.0582
$(e_3,q_3,1)$	0.0857	0.1193	0.0869	− 0.1226	0.0155	− 0.0474	0.0155	0.0762	− 0.0537
$(e_4,q_1,1)$	− 0.0426	0.0240	0.0067	− 0.0186	0.0100	− 0.0186	0.0067	0.0240	0.0100
$(e_4,q_2,1)$	− 0.0089	0.0314	− 0.0153	0.0106	0.0491	− 0.0195	− 0.0153	− 0.0132	− 0.0153
$(e_4,q_3,1)$	− 0.0102	− 0.0577	− 0.0223	0.0582	0.0049	0.0151	0.0174	− 0.0122	0.0577
$(e_5,q_1,1)$	0.0632	− 0.0284	0.0196	0.0454	0.0632	0.0055	0.0599	0.0114	− 0.0348
$(e_5,q_2,1)$	0.0024	0.0051	− 0.0087	0.0204	0.0288	0.0024	0.0126	− 0.0051	− 0.0087
$(e_5,q_3,1)$	0.0043	0.0340	0.0064	0.0340	− 0.0505	− 0.0089	− 0.0132	0.0043	− 0.0505
$(e_6,q_1,1)$	− 0.0464	0.0538	0.0262	0.0798	− 0.0227	− 0.0335	0.0605	0.0842	− 0.0152
$(e_6,q_2,1)$	0.0387	0.0279	0.0728	0.0152	0.0061	− 0.0721	0.0448	0.0721	0.0061
$(e_6,q_3,1)$	0.0152	− 0.0196	0.0632	0.0055	0.0403	0.0114	− 0.0348	− 0.0649	0.0436
$(e_7,q_1,1)$	0.0134	0.0057	0.0145	0.0211	− 0.0116	0.0029	− 0.0084	0.0018	− 0.0051
$(e_7,q_2,1)$	0.0044	0.0101	0.0140	0.0090	0.0133	0.0025	− 0.0018	0.0097	0.0067
$(e_7,q_3,1)$	− 0.0009	0.0028	0.0060	0.0013	0.0019	0.0050	0.0070	0.0049	0.0013
$(e_8,q_1,1)$	0.0202	0.0281	0.0068	0.0126	0.0291	0.0179	0.0126	0.0194	0.0061
$(e_8,q_2,1)$	0.0070	− 0.0028	0.0022	0.0050	0.0070	0.0045	0.0013	0.0049	0.0028
$(e_8,q_3,1)$	0.0134	0.0095	0.0218	0.0046	− 0.0211	− 0.0134	0.0101	0.0027	0.0038
$(e_9,q_1,1)$	0.0091	0.0269	0.0433	0.0307	0.0131	0.0421	0.0307	0.0055	0.0167
$(e_9,q_2,1)$	0.0388	− 0.0174	0.0577	0.0049	0.0562	0.0358	0.0174	0.0532	0.0483
$(e_9,q_3,1)$	0.0224	0.0046	0.0064	0.0194	− 0.0140	0.0360	0.0049	0.0148	− 0.0243
$(e_{10},q_1,1)$	− 0.0089	0.0358	0.0505	0.0043	0.0314	0.0089	0.0146	0.0153	0.0195
$(e_{10},q_2,1)$	0.0196	0.0436	0.0055	0.0114	0.0403	0.0655	− 0.0284	− 0.0649	0.0403
$(e_{10},q_3,1)$	0.0281	− 0.0155	− 0.0051	0.0051	− 0.0288	0.0087	− 0.0061	0.0291	0.0051
$(e_{11},q_1,1)$	0.0983	0.0085	0.0178	− 0.0542	0.0442	0.0213	− 0.0305	− 0.0265	− 0.0391
$(e_{11},q_2,1)$	− 0.0865	0.0165	0.0582	− 0.0283	0.0582	0.0283	− 0.0220	0.0655	− 0.0503
$(e_{11},q_3,1)$	− 0.0530	− 0.0735	0.0921	0.1383	− 0.0289	0.0116	0.0600	− 0.1370	0.0958
$(e_{12},q_1,1)$	0.0112	− 0.0051	− 0.0112	0.0155	− 0.0112	0.0076	0.0291	− 0.0051	0.0204
$(e_{12},q_2,1)$	0.0772	0.0493	0.0100	0.0534	− 0.0426	0.0167	0.0732	0.0100	− 0.0793
$(e_{12},q_3,1)$	0.0262	0.0538	0.0109	− 0.0842	0.0613	0.0227	− 0.0798	− 0.0152	0.0538
${\mathfrak {a}}_j=\sum _i p_{ij}$	${\mathfrak {a}}_1=0.5002$	${\mathfrak {a}}_2=0.4537$	${\mathfrak {a}}_3=1.0292$	${\mathfrak {a}}_4=0.4897$	${\mathfrak {a}}_5=0.3280$	${\mathfrak {a}}_6=0.1528$	${\mathfrak {a}}_7=0.5669$	${\mathfrak {a}}_8=0.3792$	${\mathfrak {a}}_9=0.2775$

The weighted disagree score table is displayed by Table 26.

**Table 26:** Weighted disagree-score table of FFSES $(\lambda ,A)_0$

$(\lambda ,A)_0$	$p_1$	$p_2$	$p_3$	$p_4$	$p_5$	$p_6$	$p_7$	$p_8$	$p_9$
$(e_1,q_1,0)$	0.0243	0.0031	0	0.0095	0.0360	0.0064	0.0046	− 0.0351	0.0256
$(e_1,q_2,0)$	0.0312	0.0327	0.0419	0.0727	− 0.0327	0.1082	0.0092	0.1053	0.0672
$(e_1,q_3,0)$	0.0448	0.0316	0.0728	0.0152	− 0.0702	− 0.0448	0.0335	0.0091	0.0127
$(e_2,q_1,0)$	0.0056	0.0042	0.0063	0.0146	0.0044	0.0097	0.0146	− 0.0030	0.0140
$(e_2,q_2,0)$	0.0089	0.0064	− 0.0195	− 0.0271	0.0340	0.0510	− 0.0106	0.0043	0.0221
$(e_2,q_3,0)$	0.0360	0.0252	0.0224	0.0095	0.0109	0.0064	0.0046	0.0224	0.0046
$(e_3,q_1,0)$	− 0.1615	0.1175	0.0435	− 0.1530	− 0.0292	0.1030	0.1674	− 0.0727	− 0.1658
$(e_3,q_2,0)$	0.0283	0.0630	0.0165	0.0118	− 0.0363	0.0079	0.0165	0.0198	0.0411
$(e_3,q_3,0)$	0.0762	− 0.0216	0.0762	0.0371	0.1238	0.0762	0.0155	0.0869	0.0258
$(e_4,q_1,0)$	0.0307	− 0.0240	0.0562	0.0732	0.0801	0.0140	0.0801	0.0100	0.0732
$(e_4,q_2,0)$	0.0505	0.0221	0.0106	− 0.0153	− 0.0132	− 0.0195	0.0082	0.0089	− 0.0505
$(e_4,q_3,0)$	− 0.0223	0.0582	− 0.0577	0.0049	0.0151	0.0577	− 0.0102	0.0174	− 0.0122
$(e_5,q_1,0)$	− 0.0284	0.0196	0.0454	0.0632	0.0055	0.0599	0.0114	− 0.0348	0.0632
$(e_5,q_2,0)$	0.0024	0.0051	− 0.0087	0.0204	0.0288	0.0024	0.0126	− 0.0051	− 0.0087
$(e_5,q_3,0)$	− 0.0089	− 0.0132	0.0043	− 0.0505	0.0043	0.0340	0.0064	0.0340	− 0.0505
$(e_6,q_1,0)$	− 0.0464	0.0538	0.0262	0.0798	− 0.0227	− 0.0335	0.0605	0.0842	− 0.0152
$(e_6,q_2,0)$	0.0721	0.0387	0.0279	0.0728	0.0152	0.0061	0.0448	0.0061	0.0702
$(e_6,q_3,0)$	− 0.0348	0.0152	− 0.0196	0.0632	0.0055	0.0403	0.0114	− 0.0649	0.0436
$(e_7,q_1,0)$	− 0.0057	0.0029	0.0134	0.0057	0.0145	0.0018	− 0.0051	0.0211	− 0.0084
$(e_7,q_2,0)$	0.0090	0.0133	0.0067	0.0044	0.0101	0.0140	0.0025	− 0.0018	0.0097
$(e_7,q_3,0)$	0.0019	0.0050	− 0.0009	0.0028	0.0060	0.0013	0.0070	0.0049	0.0013
$(e_8,q_1,0)$	0.0291	0.0179	0.0126	0.0194	0.0061	0.0202	0.0281	0.0068	0.0126
$(e_8,q_2,0)$	0.0070	0.0028	0.0045	0.0070	− 0.0028	0.0022	0.0050	0.0013	0.0049
$(e_8,q_3,0)$	0.0101	0.0027	0.0038	− 0.0211	− 0.0134	0.0134	0.0095	0.0218	0.0046
$(e_9,q_1,0)$	0.0433	0.0307	0.0131	0.0421	0.0307	0.0055	0.0167	0.0091	0.0269
$(e_9,q_2,0)$	0.0358	0.0174	0.0532	0.0483	0.0388	− 0.0174	0.0577	0.0049	0.0562
$(e_9,q_3,0)$	0.0046	0.0360	0.0049	0.0148	− 0.0243	− 0.0140	0.0109	− 0.0109	0.0360
$(e_{10},q_1,0)$	0.0340	− 0.0271	0.0106	0.0466	0.0064	− 0.0505	0.0491	− 0.0195	0.0153
$(e_{10},q_2,0)$	0.0196	0.0436	0.0055	0.0114	0.0403	0.0655	− 0.0284	− 0.0649	0.0403
$(e_{10},q_3,0)$	− 0.0288	− 0.0087	0	0	0.0179	0	0.0194	− 0.0288	0.0179
$(e_{11},q_1,0)$	0	0	0.0542	0.0127	− 0.0265	0.0291	− 0.0085	0.0305	0.0085
$(e_{11},q_2,0)$	0	− 0.0118	0	− 0.0865	− 0.0079	0.0411	− 0.0937	0.0220	0.0118
$(e_{11},q_3,0)$	0.0116	− 0.0359	0.0851	− 0.0530	− 0.0241	− 0.0600	− 0.1264	− 0.1148	− 0.0735
$(e_{12},q_1,0)$	0.0179	0.0291	− 0.0126	0.0051	0.0036	− 0.0112	− 0.0155	0.0061	0.0126
$(e_{12},q_2,0)$	− 0.0772	0.0562	0.0208	− 0.0732	0.0140	− 0.0240	0.0493	0.0307	− 0.0140
$(e_{12},q_3,0)$	0.0250	0.0073	0	0.0227	0.0865	-0.0109	0.0582	0.0402	0.0874
${\mathfrak {b}}_j=\sum _i p_{ij}$	${\mathfrak {b}}_1=0.2456$	${\mathfrak {b}}_2=0.6192$	${\mathfrak {b}}_3=0.6194$	${\mathfrak {b}}_4=0.3113$	${\mathfrak {b}}_5=0.3352$	${\mathfrak {b}}_6=0.4913$	${\mathfrak {b}}_7=0.5162$	${\mathfrak {b}}_8=0.1512$	${\mathfrak {b}}_9=0.4105$

Using Algorithm 2, we find the best alternative for vendor of solar panel system. Table 27 can be readily obtained from Tables 25 and 26.

**Table 27:** Final weighted score table

${\mathfrak {a}}_j=\sum _i p_{ij}$	${\mathfrak {b}}_j=\sum _i p_{ij}$	${\mathfrak {z}}_j={\mathfrak {a}}_j-{\mathfrak {b}}_j$
${\mathfrak {a}}_1=0.5002$	${\mathfrak {b}}_1=0.2456$	${\mathfrak {z}}_1=0.2546$
${\mathfrak {a}}_2=0.4537$	${\mathfrak {b}}_2=0.6192$	${\mathfrak {z}}_2=-0.1655$
${\mathfrak {a}}_3=1.0292$	${\mathfrak {b}}_3=0.6194$	${\mathfrak {z}}_3=0.4098$
${\mathfrak {a}}_4=0.4897$	${\mathfrak {b}}_4=0.3113$	${\mathfrak {z}}_4=0.1784$
${\mathfrak {a}}_5=0.3280$	${\mathfrak {b}}_5=0.3352$	${\mathfrak {z}}_5=-0.0072$
${\mathfrak {a}}_6=0.1528$	${\mathfrak {b}}_6=0.4913$	${\mathfrak {z}}_6=-0.3385$
${\mathfrak {a}}_7=0.5669$	${\mathfrak {b}}_7=0.5162$	${\mathfrak {z}}_7=0.0507$
${\mathfrak {a}}_8=0.3792$	${\mathfrak {b}}_8=0.1512$	${\mathfrak {z}}_8=0.228$
${\mathfrak {a}}_9=0.2775$	${\mathfrak {b}}_9=0.410$	${\mathfrak {z}}_9=-0.1325$

From the above Table 27, one can easily see that $\max {\mathfrak {z}}_j={\mathfrak {z}}_3$, so the alternative $p_3$ will be selected as best brand to purchase solar panel system for the vendor.

## Sensitivity analysis

In this section, we compare the proposed model with certain existing models to highlight its advantages and limitations.

### Advantages of the model initiated in this work

Several domains of real-life, including science and technology, contain uncertainty due to imperfect or unknown information in different epistemic situations. Until less than a century ago, probability theory—which is originally based on principles of Aristotle’s bi-valued logic—used to be the unique tool available to the experts for the modelization of uncertainty. However, different real phenomena in nature are an evidence of the existence of multiple kinds of uncertainties which cannot be dealt properly with classical mathematical tools. To put a simple example, the term ‘frosty’ means a certain range of cold temperatures, which may vary in different circumstances; certainly, the term lacks a sharp, definite boundary. The representation of a system with these types of terms contains a class of uncertainty that is called fuzziness. To address these issues, a number of researchers had proposed different approaches earlier, like multi-valued logic that had been studied in the 1920’s by Polish logicians Łukasiewicz, Tarski and others. But fuzzy set theory (Zadeh 1965) gave origin to fuzzy logic and allowed to approach uncertainly defined situations competently. The theory of fuzzy sets opened the door to the construction of mathematical solutions of problems stated in a natural language, in cases where traditional tools fail to apply. Zadeh’s remarkable idea has found many applications in several fields, inclusive of decision-making. As is well known, fuzzy sets give the degree of membership of an element in a given set (and it is implicitly assumed that its nonmembership degree equals one minus its degree of membership). In some situations however, there are separate evaluations of membership and nonmembership degrees. When the sum of both degrees is strictly smaller than 1, then the practitioner must allocate some hesitation; and this circumstance cannot be explained by means of fuzzy set theory. For this reason IFSs were initiated by Atanassov. Later on, both PFSs and FFSs were introduced for the purpose of including additional uncertain problems into a common theoretical setting.

The development of new forms of vague and uncertain informational models for group decision-making is soaring. Along the past few decades, a number of hybrid models have been reported towards this worthy topic, including different hybrid structures of fuzzy, intuitionistic fuzzy and Pythagorean fuzzy theories. There are various phenomena, which cannot be well explained via the above-mentioned existing models. In practical decision-making problems, the decision makers (or experts) may indicate their selections regarding the degree of an alternative about a criterion fulfilling the condition that the sum of the belongingness degree and non-belongingness degree or their sum of squares may be greater than 1. For example, let us suppose that some motivational scholars are enduing their opinions about modern vs. traditional societies in order to point out differences between them, or to mention the merits of one over the other. Consider the next situation: half of them give their opinion (degree) in favor of modern society which is 0.83, and the remaining half provide their opinion in favor of traditional society with a degree of 0.65. Clearly, $0.83^2 +0.65^2=1.11\nless 1$ but $0.83^3 +0.65^3=0.85< 1$. Notice that the experts’ selections in such cases cannot be suitably described by the recourse to IFSs or PFSs. It is in these situations that the FFS model acts more effectively, as it widens the space of uncertain data and relaxes the condition of IFSs and PFSs on the belongingness and non-belongingness degrees.

With the invention of the FFS model which is a direct generalization of fuzzy, intuitionistic fuzzy and Pythagorean fuzzy models, a lot of researchers were attracted towards this worthy model and its hybridizations in order to deal with different daily-life scenarios more adequately. The soft expert set model and its fuzzy version have been produced for group decision-making purposes. Our developed FFSES model is a mixture of FFSs and SESs which has the ability to deal with Fermatean fuzzy information comprising all the objects evaluations regarding each parameter in view of available experts independently. In other words, one can easily observe that the initiated model shares the merits of FFSs, FFSSs, SESs, FSESs, IFSESs and Pythagorean fuzzy SESs to address the two-dimensional obscure information from different experts. Moreover, the MCGDM method developed here is more reliable as compared to some existing multicriteria decision-making methods like TOPSIS and TODIM techniques, because these existing approaches fail to tackle a situation where the provided information contain separate opinions of different experts about alternatives with respect to available parameters in Fermatean fuzzy environment. These competencies of the proposed model make it unique and cogent for the representation and analysis of group decision-making situations.

### Comparison with existing models

To check the validity and performance of our developed hybrid model, here we use the same data of given application according to the hybrid model by which we have to compare our proposed model. The computed results by applying existing FSESs (Alkhazaleh and Salleh 2014) are summarized in Table 28.

**Fig. 1:**
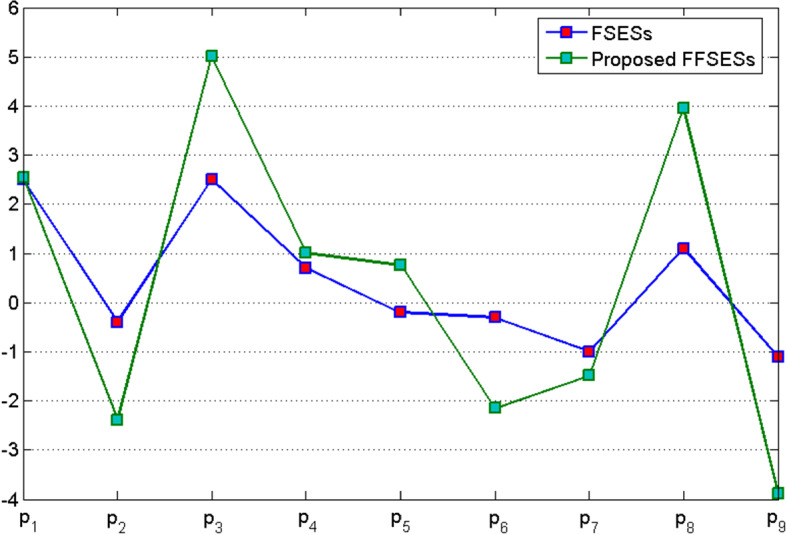
Comparison between FSESs and FFSESs in application (Sect. 4)

**Fig. 2:**
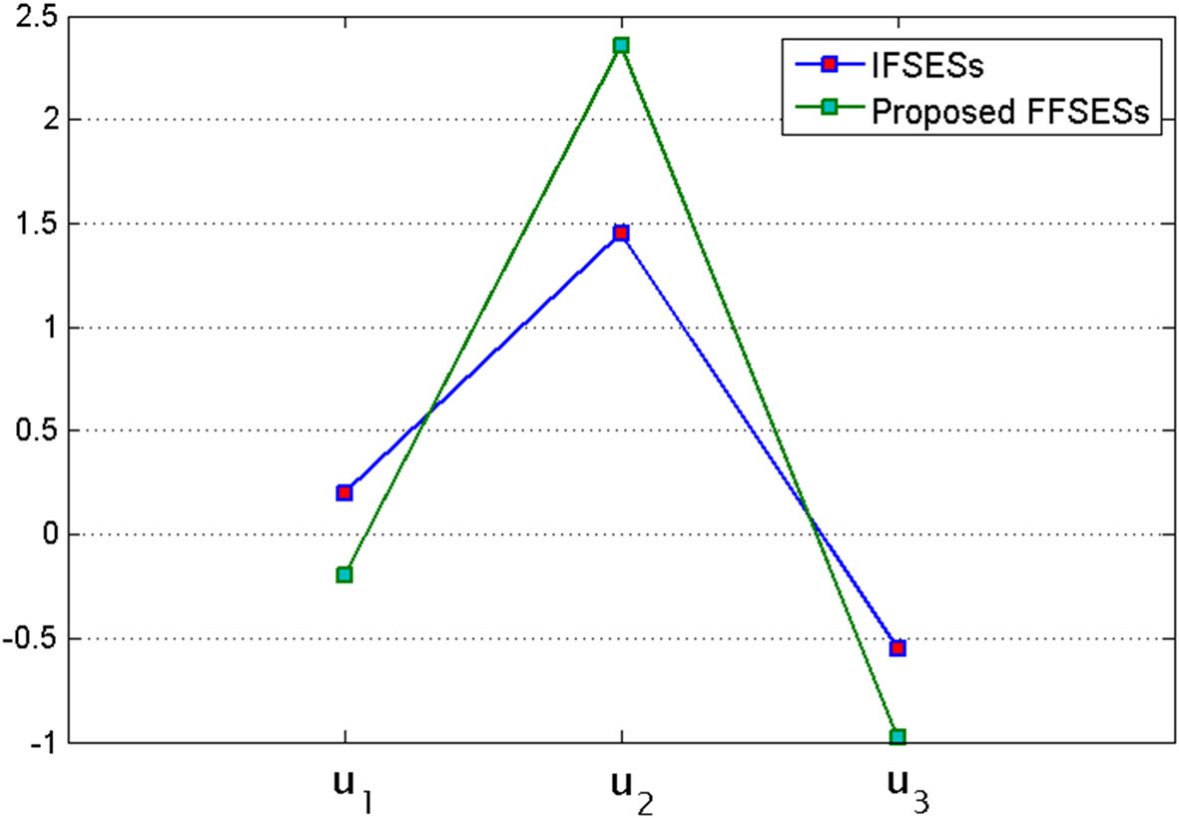
Comparison between final scores and rankings results of IFSES (Broumi and Smarandache 2015) and proposed FFSES model on the Application in (Broumi and Smarandache 2015)

It is clear from Table 28 and Fig. 1 that the most suitable options, when computed by either the FSES model (Alkhazaleh and Salleh 2014) or the proposed FFSESs, are similar. Thus our hybrid model is more reliable to solve several decision-making problems because it ranks all the alternatives of the Application (Sect. 4), but the FSES model (Alkhazaleh and Salleh 2014) fails to establish a ranking of objects $p_1$ and $p_3$ (see Table 29). The main reason behind the construction of our presented model is that the existing IFSES model (Broumi and Smarandache 2015) is able to handle situations where the sum of both membership and non-membership degrees is less than one, however it fails to cope with cases where this sum is greater than 1 at some instance. For example, if membership score 0.9 and non-membership score 0.6 are assigned, then the IFSES model is not applicable. To observe the comparison between IFSESs and the FFSES model, we have applied the methodology proposed here on the Application given in (Broumi and Smarandache 2015). From Table 30 and Fig. 2, it is clear that the optimal decision object is the same under both IFSESs (Broumi and Smarandache 2015) and the proposed FFSES model. This is why we propose the theory of FFSESs, which is more valuable than IFSESs and FSESs because its scope of application is larger than that of the existing SES models.

**Table 28:** Comparison table for the application (Sect. 4)

Objects	FSESs (Alkhazaleh and Salleh 2014)	Proposed FFSESs
$p_1$	2.5	2.555
$p_2$	− 0.4	− 2.388
$p_3$	2.5	5.015
$p_4$	0.7	1.004
$p_5$	− 0.2	0.573
$p_6$	− 0.3	− 2.151
$p_7$	− 1	− 1.484
$p_8$	1.1	3.952
$p_9$	− 1.1	− 3.886

**Table 29:** Comparison between rankings results of FSES and proposed FFSES model on the application (Sect. 4)

Models	Ranking order	Best option
FSESs (Alkhazaleh and Salleh 2014)	$p_{3}=p_{1}>p_{8}>p_{4}>p_5>p_6>p_{2}>p_7>p_{9}$	$p_3$
Proposed FFSESs	$p_{3}>p_{8}>p_1>p_{4}>p_5>p_7>p_{6}>p_2>p_{9}$	$p_3$

**Table 30:** Comparison between final scores and rankings results of IFSES (Broumi and Smarandache 2015) and proposed FFSES model on the Application in (Broumi and Smarandache 2015)

Models	$u_1$	$u_2$	$u_3$	Ranking order	Best option
IFSESs (Broumi and Smarandache 2015)	0.20	1.45	− 0.55	$u_{2}>u_{1}>u_{3}$	$u_2$
Proposed FFSESs	− 0.20	2.36	− 0.98	$u_{2}>u_{1}>u_{3}$	$u_2$

### Limitations of the model initiated in this paper

Some limitations of the proposed model which we have observed during its analysis are:

- The competency of the proposed model is limited to address the two-dimensional obscure information, that is, it fails if in a situation, we consider membership value 0.7 and non-membership value 0.9, since $0.7^3+0.9^3=1.072\nless 1$.
- Another limitation of the proposed hybrid model is the occurrence of large sets of alternatives, parameters and experts in several MCGDM problems, because the computational speed may be handicapped by the increase in available information. Like proposed model there are several existing mathematical tools involving this deficiency which can be easily control with a suitable code of developed method via different mathematical software such as MATLAB, MAPLE, etc.
- Moreover, there may be observed a variation in the ranking order of optimal or sub-optimal objects when any existing parameters (or objects) are removed or new parameters (or objects) are added in a MCGDM problem. It happens due to the autonomous behavior of objects and parameters.

## Conclusions and future directions

In this research article we have developed a novel hybrid model called FFSES model. It stems from a suitable combination of FFSs and SESs. Additionally, we have describe our model with some numerical examples. Furthermore, we have demonstrated some fundamental properties concerning operations like complement, union, intersection, the OR operation and the AND operation. We have also explored a practical application of the corresponding MCGDM methodology (that concerns the selection of a better brand of solar panel system). In fact, we have provided two algorithms that ensure the productiveness and authenticity of the model that we have introduced. Lastly, we have performed a comparison with two existing mathematical tools, that is, the FSES model (Alkhazaleh and Salleh 2014), and the IFSES model (Broumi and Smarandache 2015).

The results presented in this article have important implications for the operations strategy and supply chain management research. The analysis and results presented above have demonstrated that the proposed methodology is a useful and practical method for carrying out supplier ratings for solar panel systems.

In the future, the group decision-making approach that we have introduced in this article can be generalized to deal with other MCGDM problems more precisely. Such problems include supplier selection, employee selection or promotion, as well as several other domains of science and technology. Thus we shall work towards the extension of this research work in the following settings:

1. The ELECTRE I and TOPSIS methods can be combined with Fermatean fuzzy soft expert sets to handle Fermatean fuzzy information in case of multiple experts, like the TOPSIS method for Fermatean fuzzy soft sets (Salsabeela and John 2021).
2. The interval-valued Fermatean fuzzy sets (Jeevaraj 2021) can be combined with soft expert sets (Alkhazaleh and Salleh 2011) to construct a new model called interval-valued Fermatean fuzzy soft expert sets which is a natural interval-valued extension of Fermatean fuzzy soft expert sets.
3. To deal with more generalized Fermatean fuzzy information based on multinary graded evaluations, another extension of fuzzy *N*-soft expert sets (Ali and Akram 2020) or the proposed Fermatean fuzzy soft expert set model called Fermatean fuzzy *N*-soft expert sets can be constructed, This arises from the combination of Fermatean fuzzy sets with *N*-soft expert sets (Ali and Akram 2020).
